# Macrophages originated IL-33/ST2 inhibits ferroptosis in endometriosis via the ATF3/SLC7A11 axis

**DOI:** 10.1038/s41419-023-06182-4

**Published:** 2023-10-11

**Authors:** Qiong Wu, Zongwen Liang, Jing Jiang, Xiaoming Feng, Jinming Liu, Zongfeng Zhang, Honglin Wang, Ning Wang, Yanling Gou, Zhi Li, Yingying Cao

**Affiliations:** 1 Department of Obstetrics and Gynecology, Second Affiliated Hospital of Harbin Medical University, 150086 Harbin, China; 2 Academy of Agriculture and Food Science and Technology, HeiLongJiang Agricultural Engineearing Vocational College, Harbin, China

**Keywords:** Apoptosis, Interleukins, Immunoproliferative disorders, Cell death and immune response, DNA metabolism

## Abstract

Endometriosis is a gynecological inflammatory disease that is linked with immune cells, specifically macrophages. IL-33 secreted from macrophages is known to accelerate the progression of endometriosis. The periodic and repeated bleeding that occurs in women with endometriosis leads to excess iron in the microenvironment that is conducive to ferroptosis, a process related to intracellular ROS production, lipid peroxidation and mitochondrial damage. It is suggested that eESCs may specifically be able to inhibit ferroptosis. However, it is currently unclear whether IL-33 directly regulates ferroptosis to influence the disease course in endometriosis. In this study, eESCs co-cultured with macrophages or stimulated with IL-33/ST2 were observed to have increased cell viability and migration. Additionally, IL-33/ST2 decreased intracellular iron levels and lipid peroxidation in eESCs exposed to erastin treatment. Furthermore, IL-33/ST2 treatment resulted in a notable upregulation in SLC7A11 expression in eESCs due to the downregulation of negative transcription factor ATF3, thereby suppressing ferroptosis. The P38/JNK pathway activated by IL-33/ST2 was also found to inhibit the transcription factor ATF3. Therefore, we concluded that IL-33/ST2 inhibits the ATF3-mediated reduction in SLC7A11 transcript levels via the P38/JNK pathway. The findings reveal that macrophage-derived IL-33 upregulates SLC7A11 in eESCs through the p38/JNK/ATF3 pathway, ultimately resulting in protection against ferroptosis in eESCs. Moreover, we conducted an experiment using endometriosis model mice that showed that a combination of IL-33-Ab and erastin treatment alleviated the disease, showing the promise of combining immunotherapy and ferroptosis therapy.

## Introduction

Endometriosis (EMs) is a chronic inflammatory disease that affects nearly 10% of females of reproductive age worldwide [1]. Multiple theories on the pathophysiology of endometriosis, including ectopic implantation, coelomic metaplasia, and immune factors, have been proposed to elucidate the pathophysiology of endometriosis [2]. Notable changes in the local immune microenvironment of endometriosis lesions have been detected, including the infiltration and differentiation of diverse immune cells types and the aggregation of chemokines and cytokines [3]. Macrophages are reported to be one of the predominant cells present in ectopic endometrial lesions, and interleukin-1β (IL-1β)’s release promotes ectopic endometrial stromal cells (eESCs) proliferation [4, 5]. IL-8-Ab reduced the volume of lesions and ameliorated fibrosis and adhesion in monkey endometriosis models [6]. The interplay between infiltrated immune cells and eESCs prompts genetic and epigenetic modifications in the eESCs. Subsequently, these eESCs changes cause molecular changes and dysfunction in immune cells.

Interleukin-33 (IL-33), a member of the IL-1β family, is secreted by macrophages. Previous studies have indicated that IL-33 has the potential to stimulate tumor cell proliferation and neovascularization in ovarian cancer [7]. Knockout of IL-33 in mouse endometriosis models has led to a significant decrease in endometriotic lesion volume [8]. It is also worth mentioning that IL-33 induces macrophage anti-inflammatory polarization and stimulates the formation of splenic red pulp macrophages (RPMs) that regulate erythrocyte homeostasis and support iron recycling [9, 10]. Our team has also discovered a significant promoting effect of macrophages on endometriosis [11]. We thus postulate that macrophage-derived IL-33 may regulate eESCs survival, which ultimately advances the progression of endometriosis. However, the the complete role of IL-33 in the development of endometriosis requires further exploration.

Ferroptosis is a newly discovered programmed cell death process characterized by iron-dependent accumulation of reactive oxygen species (ROS) and lipid peroxidation [12, 13]. The key molecule in ferroptosis, SLC7A11, is a component of the L-cystine/L-glutamic acid reverse transporter system (Xct) that mediates the transmembrane transport of glutamate and cysteine. Cysteine from the extracellular space triggers glutathione (GSH) synthesis, maintaining the GSH/GSSG redox balance. Glutathione peroxidase 4 (GPX4) also plays a critical role in ferroptosis by efficiently reducing lipid hydroperoxides that accumulate in the membrane of cells undergoing ferroptosis [14–16]. A study reported that the macrophages of pancreatic ductal adenocarcinoma (PDAC) patients undergo ferroptosis, which suppressed the macrophage defense response against tumor cells [17]. In endometriotic lesions, periodic bleeding and the accumulation of antiquated blood establish an iron-enriched microenvironment, and eESCs exhibit more tolerance to ferroptosis, leading researchers to hypothesize that there are certain mechanisms in eESCs that confer resistance to ferroptosis [18]. However, the specific mechanisms remain unclear.

This study explores the functional importance of ferroptosis in the pathogenesis of endometriosis. Our results reveal that the cytokine IL-33, secreted by macrophages, plays a crucial role in upregulating the expression of SLC7A11 by downregulating ATF3 in eESCs. This downregulating hinders eESCs ferroptosis and thereby facilitates the development of endometriosis. In addition, we established a mouse model of endometriosis and employed a combination treatment involving IL-33 antibodies and a ferroptosis inhibitor, erastin. This innovative approach showed that the combination of immunotherapy and ferroptosis therapy is a successful treatment for the disease.

## Results

### IL-33 from co-cultured macrophages activates ST2 in eESCs

**Fig. 1:**
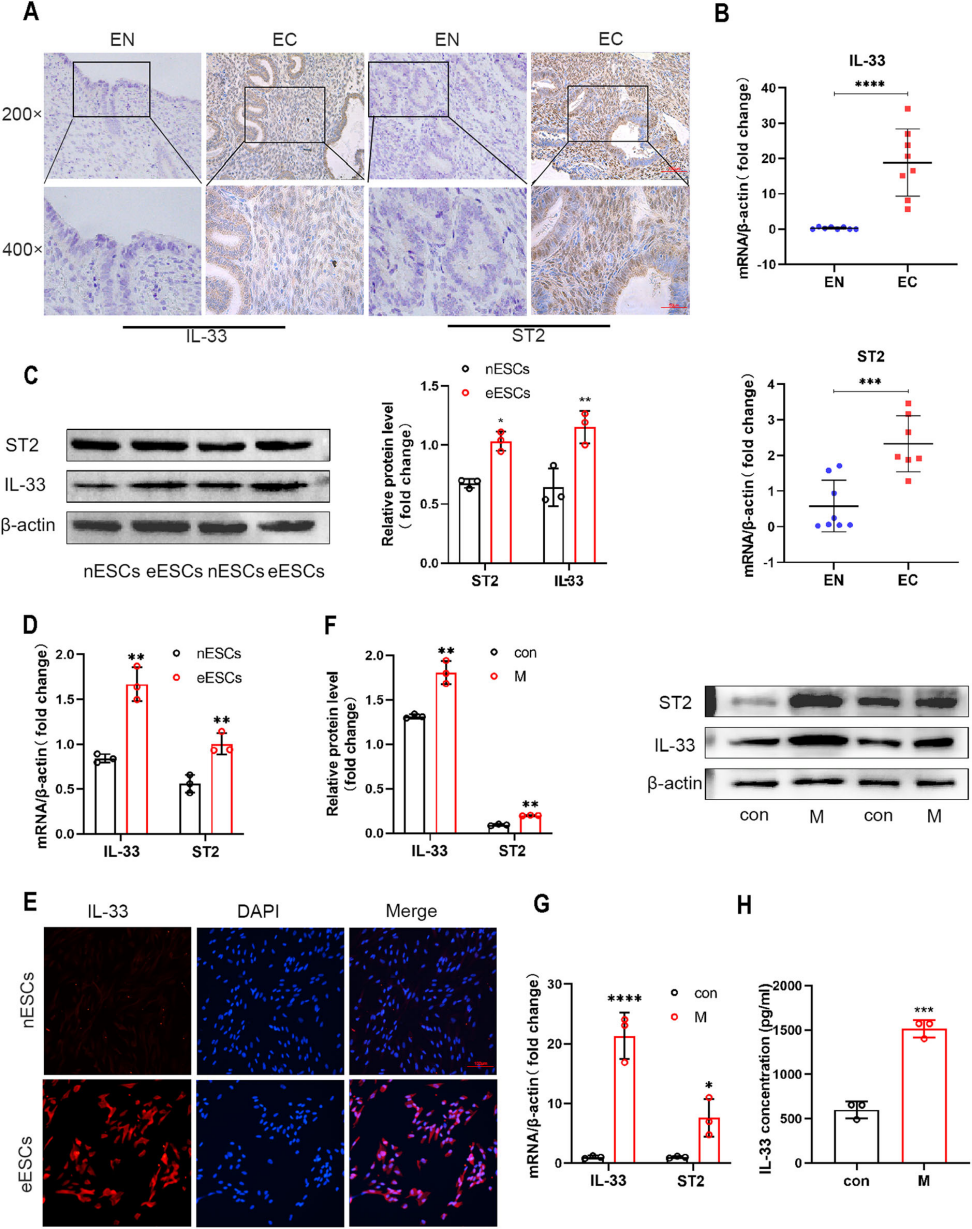
IL-33 from co-cultured macrophages activates ST2 in eESCs. **A** Representative immunohistochemical images staining with IL-33 and ST2 in normal endometrial tissue (EN) and ectopic endometriosis lesion tissue (EC). (original magnification ×200 or ×400) (*n* = 5). **B** RT-qPCR was used to determine the mRNA levels of IL-33 and ST2 in EN and EC (*n* = 8). **C** Western blot was used to detect the protein levels of IL-33 and ST2 in normal endometrial stromal cells (nESCs) and ectopic endometrial stromal cells (eESCs). **D** RT-qPCR was used to determine the mRNA levels of IL-33 and ST2 in nESCs and eESCs. **E** Representative immunofluorescence (IF) images of IL-33 (red) in nESCs and eESCs, Nuclei were stained with DAPI (blue). (original magnification ×200). **F** Western blot was used to detect the protein levels of IL-33 and ST2 in eESCs with or without macrophages co-culture treatment. **G** RT-qPCR was used to determine the mRNA levels of IL-33 and ST2 in eESCs with or without macrophages co-culture treatment. **H** ELISA analysis of IL-33 concentration in eESCs culture medium with or without macrophages co-culture treatment. Data are presented as the mean ± SD, *n* = 3 independent experiments. Statistical analysis was performed using Student’s *t* test. ****p < 0.0001, ***p < 0.001, **p < 0.01, *p < 0.05. IL-33 interleukin-33, ST2 interleukin receptor-like 1 (IL1R-L1), M macrophages, con control.

Recent studies have shown that compared to healthy females, patients with endometriosis exhibit elevated levels of interleukin-33 (IL-33) in circulation [19]. This finding piqued our curiosity about the effect of IL-33 on the progression of endometriosis. To verify the expression of IL-33 and ST2 (interleukin receptor-like 1), a widely accepted receptor of IL-33, in endometriosis (EMs) patients, we employed immunohistochemistry (IHC) assays on both normal endometrial (EN) and ectopic endometrial tissues (EC) [20]. Figure 1A illustrates that the concentration of IL-33 in EC tissues was significantly higher than that in EN tissues (Fig. 1A). The RT-qPCR results support this finding (Fig. 1B). At the cellular level, we successfully extracted, cultured and identified ectopic endometrial stromal cells (eESCs) and normal endometrial stromal cells (nESCs) (Supplementary Fig. 1). Western blot and RT-qPCR data both indicated a significant increase in the expression levels of IL-33 and ST2 in eESCs when compared with nESCs (Fig. 1C, D). The ELISA assay also demonstrated that the concentration of IL-33 was higher in eESCs cell medium than that in nESCs (Supplementary Fig. 2). The findings are further supported by results from the immunofluorescence assay (IF) performed both on eESCs and nESCs, which revealed an increase in fluorescence intensity of IL-33 and ST2 in eESCs (Fig. 1E and Supplementary Fig. 3).

The widely accepted theory suggests that the alternation of the immune micro-environment specifically macrophage differentiation, plays a critical role in the development of endometriosis [21]. Considering the mechanism responsible for the accumulation of IL-33, we note that various immune cells, including macrophages, can secrete IL-33. Based on our previous studies which indicate that macrophages are enriched around eESCs, we speculate that macrophages may be responsible for the high levels of IL-33 and ST2 in EMs [11, 22]. To test this hypothesis, we induced acute monocytic leukemia cell (THP-1 cell) differentiation using PMA (200 nM) (Supplementary Fig. 4). Western blot and RT-qPCR assays exhibited an increased IL-33 and ST2 expression levels in eESCs when they were cocultured with macrophages (Fig. 1F, G).

We then cocultured IL-33 knockdown eESCs with macrophages and measured the level of IL-33 in eESCs medium supernatant using ELISA (Supplementary Fig. 5). The concentration of IL-33 in cell supernatant of co-cultured groups still raised, suggesting that IL-33 originated from macrophages led to the formation of an environment with high IL-33 levels around eESCs. Furthermore, when comparing this in the co-culture system without IL-33 knockdown eESCs, there is no significant decrease in the concentration of IL-33 in the co-culture system supernatant. This finding suggests that the main source of IL-33 in the co-culture system is macrophages, rather than the eESCs(Fig. 1H and Supplementary Fig. 2). Luo has provided that IL-33 promotes the amplification of macrophage polarization and the production of some cytokines with anti-inflammatory effects [23]. Thus IL-33 has a dual effect, that is, it not only protects eESCs, but also promotes the polarization of anti-inflammatory macrophage sub-types. The results of these experiments reveal that macrophages induce higher IL-33 level in environment and higher ST2 expression in eESCs.

### IL-33/ST2 increased the survival rate and migration ability of eESCs

**Fig. 2:**
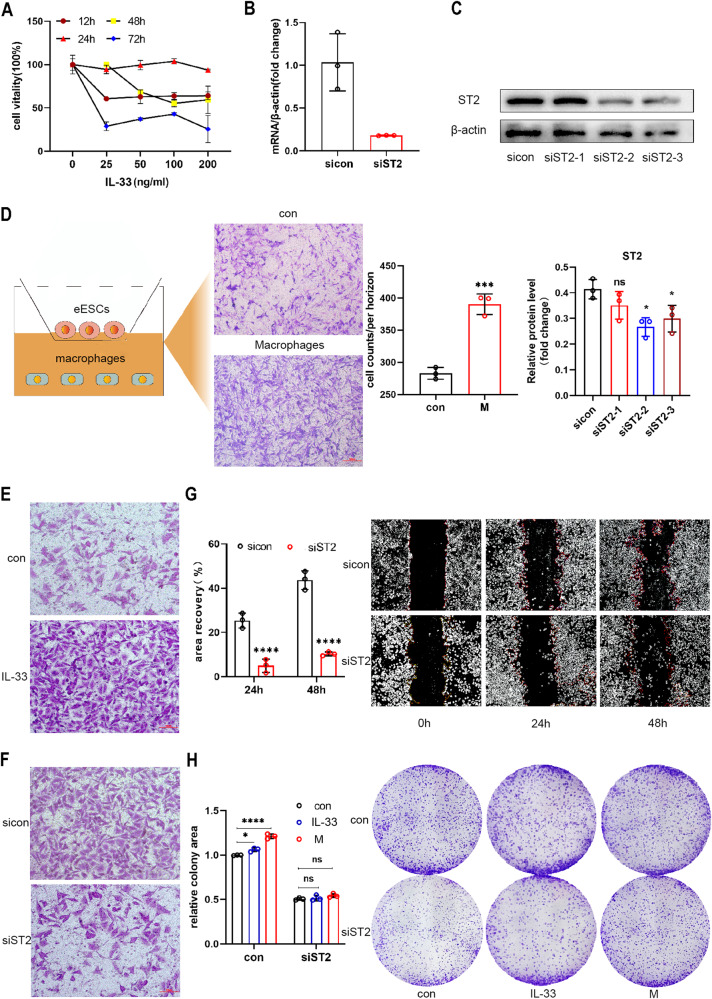
IL-33/ST2 increased the survival rate and migration ability of eESCs. **A** The eESCs were treated with specified concentrations of human recombinant IL-33 protein (rIL-33) (25, 50, 100, and 200 ng/ml) for different times (12, 24, 48, and 72 h). CCK-8 assays were performed to detect cell viability in different groups. **B** RT-qPCR was used to determine the relative levels of ST2 mRNA in eESCs transfected with siST2 (50 nM), sicon (50 nM) for 48 h. **C** Western blot was used to determine the protein levels of ST2 in eESCs transfected with siST2 (50 nM) or sicon (50 nM) for 48 h. **D** Transwell migration assay was performed to detect the migrant ability of eESCs treated with or without macrophages co-culture. Cartoon picture showed the experimental progress. (original magnification ×200). **E**, **F** Transwell migrantion assays were performed in rIL-33 (100 ng/ml) treated eESCs (**E**) and siST2 (50 nM) transfected eESCs (**F**). (original magnification ×200). **G** The wound healing assays were conducted in eESCs transfected with siST2 (50 nM) and control group. (original magnification ×40). **H** Colony formation assays were performed in siST2 (50 nM) transfected eESCs treated with rIL-33 (100 ng/ml) or macrophages co-culture. (original magnification ×40).Data are presented as the mean ± SD, *n* = 3 independent experiments. Statistical analysis was performed using Student’s *t* test (**B**–**D**, **G**) or 2-way ANOVA (**H**). ****p < 0.0001, ***p < 0.001, *p < 0.05, ns, non-significant. sicon negative control siRNA, siST2 siRNA targeting ST2, M macrophages co-culture treatment.

To elucidate the role of IL-33 secreted by macrophages on eESCs, human recombinant IL-33 protein (rIL-33) was added to the culture medium of eESCs at different concentrations (0-25-50-100-200 ng/ml). Then, we evaluated cell viability after 12, 24, 48, and 72 h to determine the optimal concentration and time parameters. Treatment with 100 ng/ml IL-33 for 24 h showed the most substantial increase in cell viability, and this concentration and time model was used in subsequent experiments (Fig. 2A). Additionally, eESCs were transfected with siRNA targeting ST2, and the efficiency of transfection was confirmed by both RT-qPCR and Western-blotting methods (Fig. 2B, C). Next, eESCs were treated with rIL-33 or cocultured with macrophages. The cell migration ability was evaluated using the transwell assay, and both treatments significantly promoted eESC migration (Fig. 2D, E and Supplementary Fig. 6). Furthermore, upon the knockdown of ST2, the migration capability of eESCs was attenuated (Fig. 2F, G and Supplementary Fig. 6). Furthermore, the cell colony formation assay showed that both rIL-33 and macrophage co-culture treatment markedly improved eESCs viability. Conversely, si-ST2 eESCs had reduced cell viability, as evidenced by the results of this assay (Fig. 2H). Collectively, these experimental findings suggest that IL-33 plays a critical role in the survival and migration of eESCs.

### IL-33/ST2 inhibited ferroptosis in eESCs

**Fig. 3:**
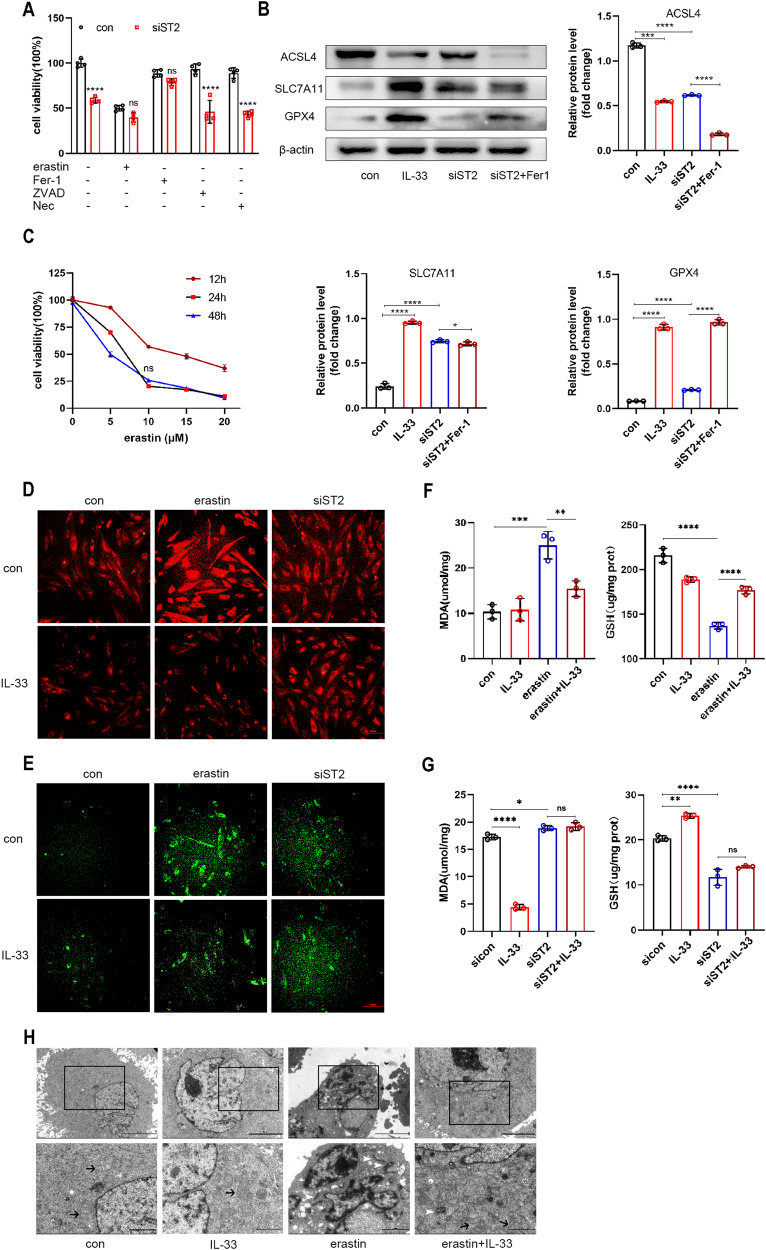
IL-33/ST2 inhibited ferroptosis in eESCs. **A** Cell viability was detected by CCK-8 assay in siST2 (50 nM) transfected eESCs with different inhibitors treatment (erasin, 10 μM; Ferrostatin-1, 1 μM; ZVAD-FMK, 10 μM; Necrostatin, 10 μM) for 24 h. **B** Western blot was used to detect the protein levels of ACSL4, SLC7A11, and GPX4 in different groups of eESCs treated with rIL-33 (100 ng/ml), siST2 (50 nM), or siST2 (50 nM)+Ferrostatin-1 (1 μM). **C** Cell viability was measured by CCK-8 assay in eESCs treated with different concentration of erasin (5, 10, 15, and 20 μM) for 12, 24, or 48 h. **D** Intracellular Fe2+ were detected by treating eESCs with 1 μM FerroOrange after indicated treatment: (a) we add erastin (10 μM) and rIL-33(100 ng/ml) into eESCs, eESCs treated with no treatment (con) and only with rIL-33 play as compared groups. (b) We added rIL-33 (100 ng/ml) into siST2 transfected eESCs, only siST2 transfected and rIL-33 addition only play as compared groups (original magnification ×200). **E** LiperFluo reagent (5 μM) were used to detect intracellular lipid peroxidation levels in eESCs with indicated treatment (same as treatment in (**D**)). (original magnification ×200). **F**, **G** The MDA levels (**F**) and GSH levels (**G**) were measured in eESCs with indicated treatment (same as treatment in (**D**)). **H** Transmission Electron Microscopy (TEM) was used to observe the morphological changes of eESCs mitochondria. black arrowheads: normal mitochondria. white arrowheads: shrunken mitochondria. The scale bar = 5.0 μm (Upper row). The scale bar = 2.0 μm (Lower row). Data are presented as the mean ± SD, *n* = 3 independent experiments. Statistical analysis was performed using Student’s *t* test (**A**) or one-way ANOVA (**B**, **F**, **G**). ****p < 0.0001, ***p < 0.001, **p < 0.01, *p < 0.05, ns non-significant, Fer-1 Ferrostatin-1, Nec Necrostatin-1, ZVAD-FMK, benzyloxycarbonyl-Val-Ala-Asp (OMe)-fluoromethylketone, ACSL4 acyl-CoA synthetase long-chain family member 4, SLC7A11 solute carrier family 7 member 11, GPX4 glutathione peroxidase 4, MDA malondialdehyde, GSH glutathione.

Endometriosis involves various forms of cell death, including apoptosis, necroptosis, autophagy, and ferroptosis [24]. To examine whether IL-33 promotes eESCs survival by regulating cell death, we compared the effects of various cell death inhibitors on si-ST2 treated eESCs. As presented in Fig. 3A, only ferrostatin-1 had a significant effect in rescuing the si-ST2-induced decline in eESCs viability, while the well-known apoptosis and necroptosis inhibitors ZVAD-FMK and necrostatin-1 showed little impact (Fig. 3A). Therefore, we presume that IL-33 may promote eESCs survival by inhibiting ferroptosis.

Next, we tested the expression levels of ferroptosis related markers in eESCs treated with rIL-33 or ST2 knockdown. The rIL-33-treated groups showed elevated levels of SLC7A11 and GPX4 but reduced levels of ACSL4 compared to those of the control group. Conversely, in the ST2 knockdown groups, and the ferroptosis inhibitor ferrostatin-1 (Fer-1) abrogated these effects (Fig. 3B).

To further explore IL-33’s role in ferroptosis progression, we treated eESCs with erastin, a powerful inducer of ferroptosis (Fig. 3C). The addition of IL-33 abrogated the increase in Fe2+ concentration triggered by erastin, thereby rescuing the cells from ferroptosis. The protective effect of IL-33 disappeared in the ST2 knockdown groups (Fig. 3D). Additionally, as ferroptosis is closely associated with lipid peroxidation, we investigated the effects of IL-33 on lipid peroxidation in eESCs. As shown in Fig. 3E, the fluorescence intensity of eESCs decreased after rIL-33 was added. In contrast, si-ST2 transfection attenuated the effect of IL-33 on reducing lipid peroxidation (Fig. 3E). The level of malonaldehyde (MDA), which is a lipid peroxidation product, increased in the erastin-treated group but was reduced in the rIL-33-treated group. Additionally, the treatment of eESCs with erastin led to the depletion the level of GSH, which is a well-known antioxidant. The addition of IL-33 restored GSH levels (Fig. 3F). However, when ST2 was knocked down in eESCs, the protective antioxidant effect of IL-33 weakened, which suggests that the presence of ST2 is necessary for IL-33 to resist ferroptosis, regulating the MDA and GSH levels in eESCs (Fig. 3G).

Ferroptosis is closely linked to mitochondrial dysfunction and reduced mitochondrial redox capacity. Through transmission electron microscopy (TEM), we observed that erastin-treated eESCs exhibited significant mitochondrial structural alterations, such as atrophy and increased membrane density, whereas IL-33 protected the mitochondrial structure from ferroptosis-induced damage (Fig. 3H). Overall, the results demonstrated the antiferroptotic role of IL-33/ST2 in eESCs.

### IL-33/ST2 inhibited ferroptosis by regulating SLC7A11 expression in eESCs

**Fig. 4:**
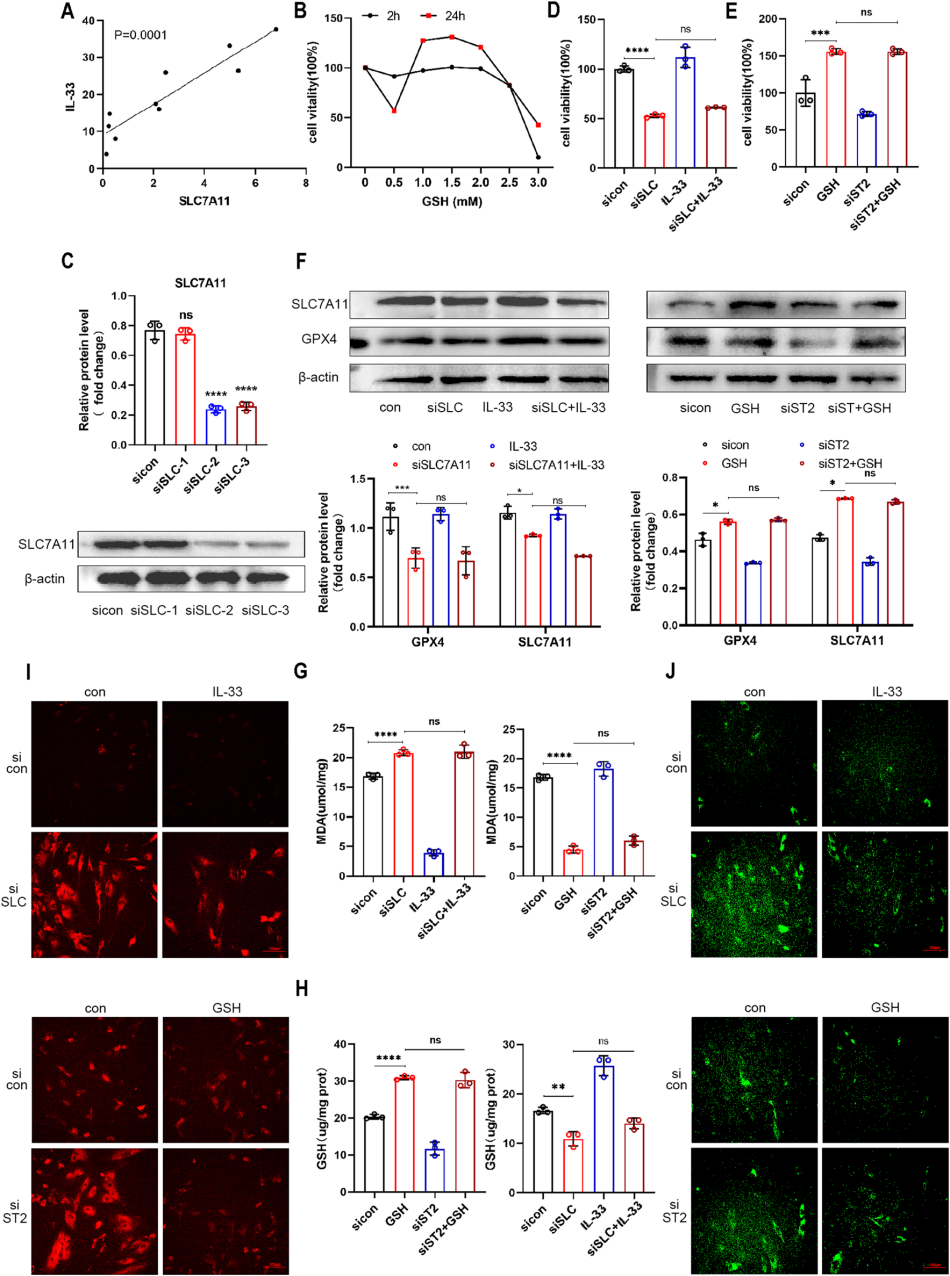
IL-33/ST2 inhibited ferroptosis by regulating SLC7A11 expression in eESCs. **A** Pearson’s test was used to analyze the relationship between the levels of IL-33 and SLC7A11 mRNA in EC tissues (*n* = 8). **B** EESCs were treated with specified concentrations of GSH (0.5, 1.0, 1.5, 2.0, 2.5, and 3.0 mM) for 2 or 24 h. CCK-8 assay was used to detect cell viability. **C** Western blot was used to determine the efficiency of siRNA-mediated knockdown of SLC7A11 in eESCs (50 nM). **D**–**J** EESCs underwent indicated treatment: rIL-33(100 ng/ml) and/or transfection with siSLC7A11 (50 nM); GSH (1.5 mM) and/or transfection with siST2 (50 nM). **D**, **E** Cell viability was detected by CCK-8 assay. **F** Western blot was used to detect the protein levels of SLC7A11, and GPX4 in eESCs. **G**, **H** Determinations of intracellular MDA (**G**) and GSH levels (**H**). **I** FerroOrange reagent (1 μM) was used to measure intracellular Fe2+concentration. (original magnification ×200). **J** LiperFluo reagent (5 μM) was used to determine intracellular lipid peroxidation level. (original magnification ×200). Data are presented as the mean ± SD, *n* = 3 independent experiments. Statistical analysis was performed using Student’s *t* test (**C**) or one-way ANOVA (**D**, **E**, **G**, **H**) or Two-way ANOVA (**F**). ****p < 0.0001, ***p < 0.001, **p < 0.01, *p < 0.05, ns non-significant, siSLC siRNA targeting SLC7A11.

To investigate the underlying mechanism by which IL-33 inhibits ferroptosis in eESCs, we conducted correlation analyses between IL-33 and several ferroptosis marker molecules. Pearson’s correlation coefficient confirmed a statistically significant association between IL-33 and SLC7A11 expression (Fig. 4A). Although we also observed a significant correlation between IL-33 and GPX4 expression (Supplementary Fig. 7), studies previously have revealed GPX4 as a downstream component of the SLC7A11 pathway during the ferroptosis process [25]. Therefore, we focused on investigating the function of SLC7A11 in our subsequent studies. The expression of SLC7A11 was notably upregulated following treatment with rIL-33 (Fig. 3D). Given that SLC7A11 plays a crucial role in forming the glutamate cysteine transporter system (Xct), which is responsible for GSH synthesis, we supplemented eESCs with GSH to overexpress SLC7A11 (Fig. 4B).

To further investigate the role that SLC7A11 plays in the process by which IL-33 inhibits ferroptosis, we knocked down SLC7A11 in eESCs (Fig. 4C and Supplementary Fig. 8). Consequently, cell viability was considerably reduced, and rIL-33 was unable to rescue the declining cell viability (Fig. 4D). However, the addition of GSH rescued the reduced cell viability induced by si-ST2 (Fig. 4E). These data suggested that SLC7A11 plays a downstream role in the IL-33 pathway. In support of these findings, Western blot results showed decreased protein level of GPX4 in the si-SLC7A11 group, further confirming the involvement of SLC7A11 in the IL-33-mediated suppression of ferroptosis. Additionally, Western blot analysis indicated that GSH could prevent ferroptosis in eESCs by downregulating the si-ST2 induced expression of ferroptosis marker molecules (Fig. 4F). These results were further substantiated through the evaluation of MDA content, GSH levels, intracellular Fe2+ concentration, and lipid peroxidation (Fig. 4G–J). Overall, our results demonstrated that IL-33/ST2 inhibits ferroptosis by modulating SLC7A11 expression in eESCs.

### IL-33/ST2 upregulated SLC7A11 by regulating ATF3

**Fig. 5:**
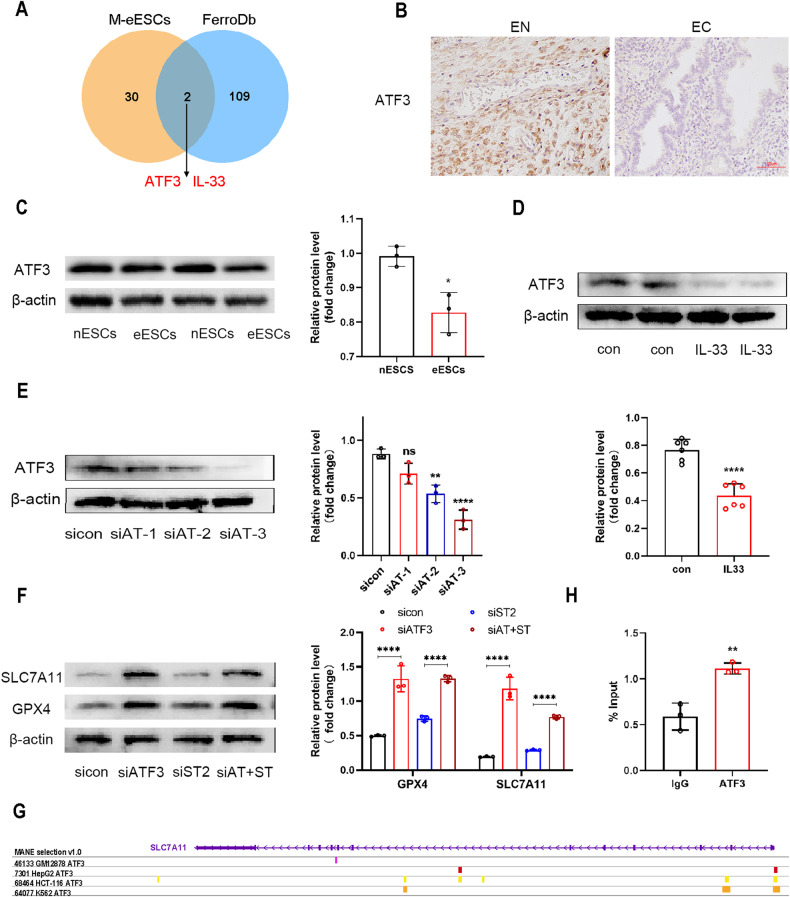
IL-33/ST2 upregulated SLC7A11 by regulating ATF3. **A** Venn graph showed the intersection of the differential expression gene in Macrophage co-culture treated eESCs and Ferroptosis marker (data originated from GSE 19834 and FerroDb). **B** Representative immunohistochemical images staining with ATF3 in normal endometrial tissue (EN) (*n* = 8) and ectopic endometriosis lesion tissue (EC) (*n* = 8) (original magnification ×200). **C** Western blot was used to detect the protein levels of ATF3 in nESCs and eESCs. **D** Western blot was used to detect the protein levels of ATF3 in eESCs treated with or without rIL-33 (100 ng/ml). **E** Western blot was used to determine the knockdown efficiency of si-ATF3 in eESCs. **F** Western blot was used to detect the protein levels of SLC7A11 and GPX4 in different groups: sicontrol (50 nM), siATF3 (50 nM), siST2 (50 nM) and double transfection of siATF3 (50 nM) and siST2 (50 nM). **G** The ChIP-seq data previously reported were reanalyzed.(GSM1917770, ENCSR632DCH_2, GSM803508, GSM803503). **H** Chromatin immunoprecipitation assay (CHIP) was used to verify the binding region of ATF3 and SLC7A11 promoter. Data are presented as the mean ± SD, *n* = 3 independent experiments. Statistical analysis was performed using Student’s *t* test. ****p < 0.0001, **p < 0.01, *p < 0.05. ATF3 activating transcription factor, siATF3 siRNA targeting ATF3, siAT+siST siRNA targeting ATF and siRNA targeting ST2.

To explore the mechanism responsible for the IL-33/ST2-mediated activation of SLC7A11 in endometriosis, we first identified differentially expressed genes (DEGs) using an endometriosis dataset obtained from the GEO database (GSE19834). This dataset compared the gene expression profiles of eESCs cultured alone and eESCs co-cultured with macrophages. A Venn diagram analysis was then constructed to identify ferroptosis marker genes (listed in the FerroDb database) among the identified DEGs. This comparison revealed IL-33 and ATF3 as two significant regulators of ferroptosis in endometriosis (Fig. 5A). ATF3 is a negative transforming factor involved in various diseases [26, 27]. ATF3 was found to regulate SLC7A11 expression [28]. Hence, we aimed to test whether ATF3 participates in the IL-33-mediated upregulation of SLC7A11 in endometriosis. Our findings illustrated that the level of ATF3 was lower in EC tissues than in EN tissues (Fig. 5B), and the ATF3 protein level in eESCs was also lower than that in nESCs (Fig. 5C). Interestingly, when rIL-33 was added to eESCs, the protein level of ATF3 was further reduced. This finding indicates that IL-33 negatively regulates ATF3 expression (Fig. 5D).

To determine the precise role that ATF3 plays in the regulatory effect of IL-33 on SLC7A11, we conducted single knockdown of ATF3, and dual knockdowns of ST2 and ATF3 in eESCs (Fig. 5E and Supplementary Fig. 9). The Western blot results demonstrated that si-ATF3 was effective in reversing the decrease in SLC7A11 and GPX4 expression levels induced by si-ST2 (Fig. 5F). Next, ChIP-seq analysis revealed the substantial presence of ATF3-binding peaks in the promoter sites of SLC7A11. Such findings indicate that ATF3 plays a crucial role in controlling the transcription of SLC7A11 by binding with high affinity to its promoter (data derived from GSM1917770, ENCSR632DCH_2, GSM803508, and GSM803503) (Fig. 5G). We then performed chromatin immunoprecipitation assays (ChIP) to confirm the direct binding of ATF3 and the SLC7A11 promoter (Fig. 5H). Collectively, we have confirmed that ATF3 serves as a negative transcription factor that impedes IL-33/ST2-mediated SLC7A11 upregulation.

### IL-33/ST2 inhibited ATF3 through the P38/JNK signaling pathway

**Fig. 6:**
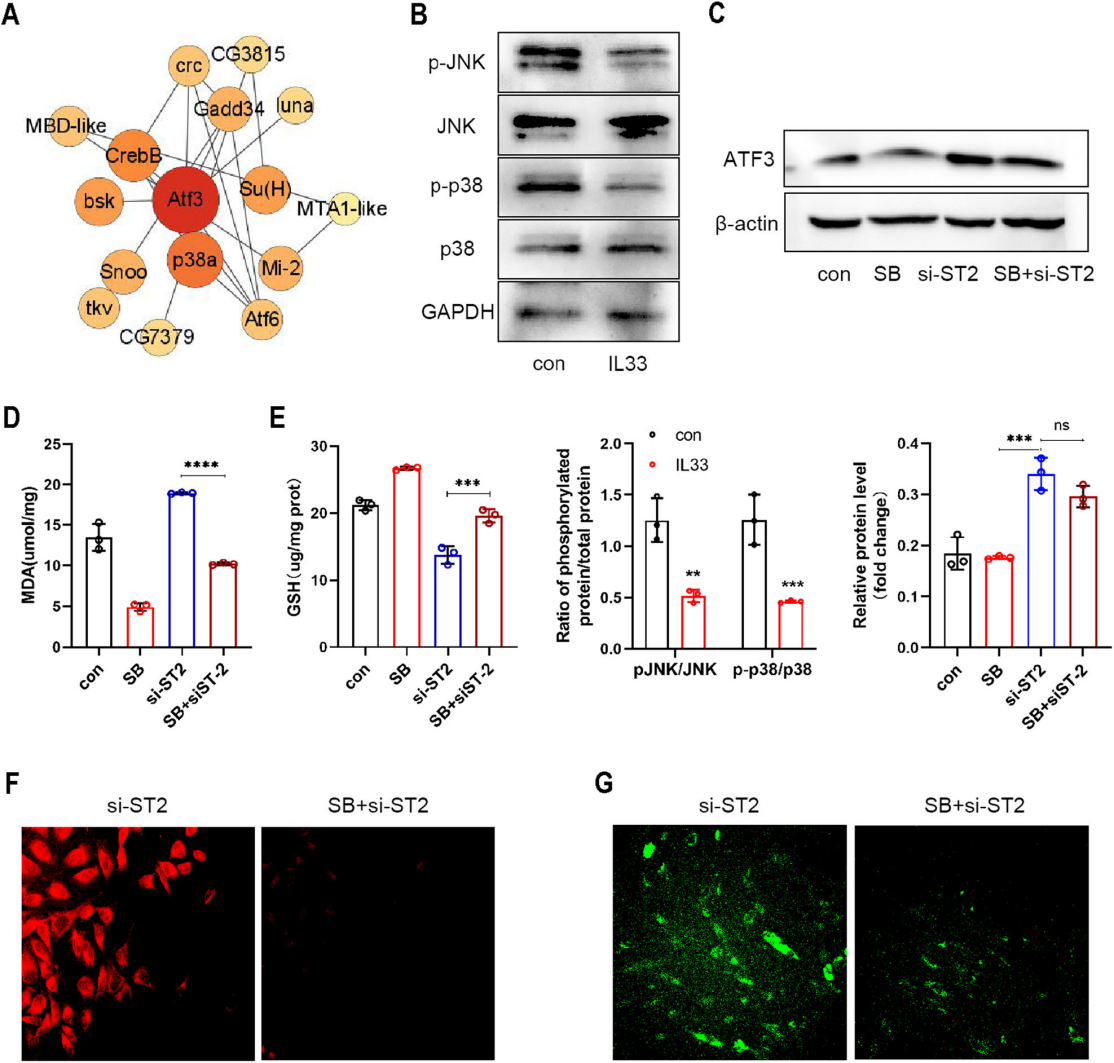
IL-33/ST2 inhibited ATF3 through the P38/JNK signaling pathway. **A** Bubble chart showed the predicted ATF3 interaction protein (data originated from String database). **B** Western blot was used to detect the protein levels of JNK, P38, phosphorylated JNK, and phosphorylated P38 in eESCs treated with or without rIL-33 (100 ng/ml). **C** Western blot was used to detect the protein levels of ATF3 in eESCs treated with siST2 (50 nM) and/or SB202190 (P38 inhibitor) (20 μM). **D**, **E** Determination of MDA (**D**) and GSH (**E**) levels in eESCs treated with the specified treatment (SB202190 (20 μM); siST2 and siST2 + SB202190 (20 μM)). **F**, **G** FerroOrange (**F**) and LiperFluo (**G**) were used to determine intracellular Fe2+ concentration and lipid peroxidation levels in siST2 transfected eESCs treated with or without SB202190 (original magnification ×200). Data are presented as the mean ± SD, *n* = 3 independent experiments. Statistical analysis was performed using one-way ANOVA. ****p < 0.0001, ***p < 0.001, **p < 0.01, ns non-significant, JNK c-Jun N-terminal protein kinase, p38 p38 MAPK p38 mitogen activated protein kinases, p-JNK phosphorylated c-Jun N-terminal protein kinase, p-p38 phosphorylated p38 mitogen activated protein kinases, GAPDH glyceraldehyde-3-phosphate dehydrogenase, SB SB202190, P38 inhibitor.

The above results indicate that IL-33/ST2 can stimulate SLC7A11 expression by downregulating ATF3. However, an important question followed is how IL-33/ST2 modulates the expression of ATF3. By analyzing the interacting protein partners of ATF3 through the STRING online database, we discovered a strong correlation between ATF3 and P38 MAPK (Fig. 6A). A study proposed that phlorofucofuroeckol A (PFF-A) has anti-cancer properties by inducing ATF3 expression via the p38 MAPK/JNK-mediated pathway in human colorectal cancer cells [29]. Further evidence indicates that stimulation of Human Keratinocytes (HaCaT) cells with thapsigargin triggers a signaling pathway that activates JNK and ATF3 activity in keratinocytes [30]. Based on this evidence, it is plausible that ATF3 may be regulated by the p38 MAPK/JNK pathway [30]. We further investigated the effects of rIL-33 on the P38 MAPK/JNK signaling pathway. Intriguingly, our findings revealed that treatment with rIL-33 suppressed the phosphorylation of both P38 MAPK and JNK (Fig. 6B). Moreover, our findings showed that a specific inhibitor of p38 MAPK phosphorylation, SB202190, elicited a similar decrease in ATF3 expression levels as that observed with IL-33 treatment. Furthermore, we did not observe significant differences in ATF3 levels in eESCs treated with SB202190. We speculate that P38 MAPK and JNK exerts their downstream effects by activating ATF3 protein or collaborating with ATF3 (Fig. 6C). Importantly, the results of tests measuring MDA content, GSH levels, intracellular Fe2+ concentration, and lipid peroxidation consistently demonstrated that SB202190 has the ability to reverse the ability of ST2 knockdown to induce ferroptosis (Fig. 6D–G). In summary, our findings suggest that IL-33/ST2 can suppress ATF3 by modulating the P38 MAPK/JNK signaling pathway, thereby upregulating SLC7A11 expression.

### Collaborative treatment with IL-33-Ab and erastin alleviated endometriosis in a mouse model

We have shown that knockdown of IL-33 inhibits ferroptosis of eESCs in vitro, so we hypothesize that IL-33-Ab can also promote ferroptosis and reduce the volume of ectopic lesions in vivo. Surprisingly, our previous results showed that erastin can significantly reduce the volume of ectopic lesions. We therefore propose a novel strategy for combining erastin with IL-33-Ab to achieve synergistic therapy. This innovative approach represents a new direction for endometriosis management.

**Fig. 7:**
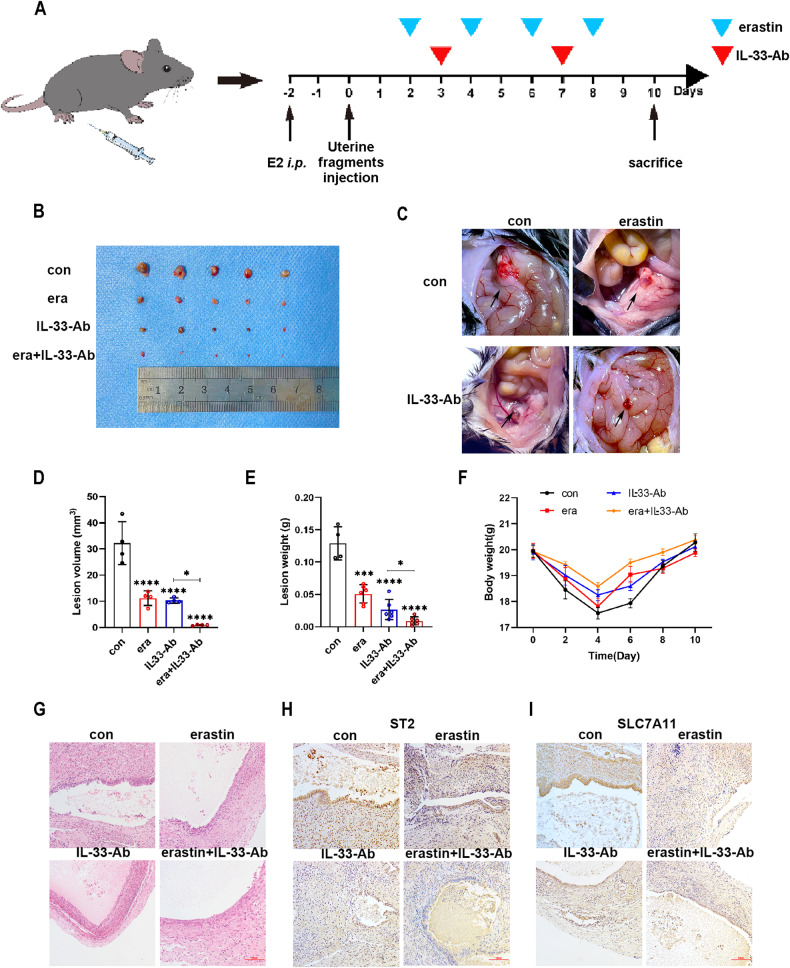
Collaborative treatment with IL-33-Ab and erastin alleviated endometriosis in a mouse model. **A** Schematic diagram showing the mouse endometriosis model establishment and therapy process. **B**, **C** Representative photo of ectopic lesions in four groups (*n* = 6) at day 10. **D**, **E** Comparison of the volume (**D**) and weight (**E**) of endometriosis ectopic lesions in four groups at day 10. **F** Line chart for the mice weight in the four groups (*n* = 6) at different time points, which showed no significant change. **G** Hematoxylin–eosin staining (H&E staining) showing that glandular and stromal structures of endometriosis ectopic lesions in four groups (original magnification ×200). **H**, **I** Representative immunohistochemical images staining with ST2 (**H**) and SLC7A11 (**I**) in four groups endometriosis ectopic lesions.(original magnification ×200). Data are presented as the mean ± SD, *n* = 3 independent experiments. Statistical analysis was performed using Two-way ANOVA (**D**) and one-way ANOVA (**E**, **F**). ****p < 0.0001, ***p < 0.001, *p < 0.05, ns non-significant, E2 17-β-estradiol-3-benzoate, i.p. intraperitoneal injection, era erastin.

To achieve this objective, we successfully established a mouse model of endometriosis and divided the mice into four groups: control, erastin, IL-33-Ab, and IL-33-Ab plus erastin, as represented in Fig. 7A. Notably, the application of both IL-33-Ab and erastin slowed the development of endometriosis. Moreover, the combination treatment was found to be more efficient in reducing the severity of the disease (Fig. 7B–E). However, it had no effect on the body weight of mice (Fig. 7F). Hematoxylin and eosin (HE) staining revealed contrasting endometrial tissue structures in the four groups (Fig. 7G). Additionally, IHC assays demonstrated a decline in the expression levels of ST2 and SLC7A11 in the IL-33-Ab and erastin treatment groups, while the combination group exhibited a more significant effect (Fig. 7H, I). By exploring the effect of IL-33-Ab and erastin on ferroptosis in endometriosis model mice, we found that they have the potential treat endometriosis. Furthermore, the combined treatment of IL-33-Ab and erastin resulted in a reinforced therapeutic effect on endometriosis model mice. This study lays the foundation for research on using this novel combination treatment of immunotherapy and ferroptosis therapy for endometriosis management.

## Discussion

Endometriosis has been found to be associated with various factors, including hereditary factors, retrograde menstruation, coelomic epithelial metaplasia, and immune factors [31]. Research has indicated that macrophages play a crucial role in promoting the colonization of endometriosis lesions by facilitating angiogenesis and matrix remodeling [32]. Our team has previously discovered that the CCL20/CCR6 signaling axis mediated by macrophages can promote the proliferation and migration of ESCs by blocking autophagic flux in endometriosis [22].

Here, we found that interleukin-33 (IL-33) is significantly highly expressed in eESCs cocultured with macrophages, contributing to eESCs survival and migration. This finding suggests that IL-33 derived from macrophages promotes the progression of endometriosis.

Different types of cell death are associated with endometriosis, such as necroptosis, autophagy, apoptosis, and ferroptosis [24]. The accumulation of endometrial debris and periodic bleeding leads to an environment with excess iron that promotes ferroptosis [33]. But it seems that eESCs are more tolerant of high iron concentrations [34]. The role of ferroptosis in endometriosis has not yet been systematically examined.

Our study revealed that Fer-1 can effectively abrogate the altered expression of ferroptosis markers induced by si-ST2. Treatment with rIL-33 also restricted ferroptosis in eESCs previously treated with erastin, as evidenced by the reduced levels of Fe2+, lipid peroxidation, and MDA, increased levels of GSH and specific mitochondrial structural changes.

In addition, we screened a screening of several molecules that were significantly altered following IL-33 treatment and identified a strong correlation between SLC7A11 and IL-33. The protective effect of IL-33 against ferroptosis was abrogated by knockout of SLC7A11. The introduction of GSH rescued eESCs from si-ST2-induced ferroptosis. Our data validated that rIL-33 upregulates the expression of SLC7A11 and blocks erastin-induced ferroptosis.

We next examined the regulators of SLC7A11. Bioinformatics analysis revealed that activating transcription factor 3 (ATF3) is one of the differentially expressed genes in eESCs cocultured with macrophages and that ATF3 plays an important role in ferroptosis. ATF3 is a transcription factor known to regulate the expression of various molecules. ATF3 deficiency has been linked to prostate tumorigenesis induced by the inhibition of Pten in mice [35]. We indeed found that IL-33 induced a significant downregulation in ATF3 expression in eESCs. Our study also revealed that the knockdown of ATF3 led to a significant upregulation in SLC7A11 expression, thereby reversing the negative effect of si-ST2 on SLC7A11. In addition, using ChIP assays, we verified the interaction of ATF3 with the SLC7A11 promoter. Taken together, our data suggest that IL-33/ST2 regulates ferroptosis by inhibiting the downregulation effect of ATF3 on SLC7A11.

Another intriguing finding of our study is the restrained activation of the p38 MAPK/JNK pathway in eESCs treated with rIL-33. Previous research has highlighted the role of p38 MAPK/JNK in upregulating ATF3 expression [36]. We also found that rIL-33 treatment had a similar inhibitory effect on ATF3 expression as treatment with the p38 MAPK inhibitor SB202190. And SB202190 treatment was able to inhibit the functions of ATF3 observed in si-ST2 eESCs. By measuring the levels of Fe2+, lipid peroxidation, MDA, and GSH, we confirmed the protective effects of SB202190 against ferroptosis triggered by si-ST2 in eESCs. In summary, our study has shed light on the mechanism of the promotive effect of IL-33/ST2 in EMs, which involves the regulation of SLC7A11 expression via the p38/JNK/ATF3 signaling pathway, thereby hindering ferroptosis.

During the progression of ferroptosis, certain molecules are released into the extracellular environment, which recruitments of immune cells. This immune cell infiltration strengthens the body’s immune defense system and has therapeutic effects on endometriosis.

Accordingly, some researchers have proposed combining immunotherapy with ferroptosis-inducing treatments as a potential approach. For instance, Niu et al. reported that ferroptosis inducers can enhance the sensitivity of “cold” tumors to immune therapy [37]. A combination of the ferroptosis inducer RSL-3 and dihydroartemisinin (DHA), an immunotherapy medicine targeting PD-L1, was designed for the treatment of pancreatic ductal adenocarcinoma (PDAC) [38].

Here, we propose a novel therapeutic approach for endometriosis that combines the ferroptosis inducer erastin and IL-33-Ab as ferroptosis immunotherapy. IL-33-Ab not only lowers the tolerance of eESCs to ferroptosis but also stimulates macrophage polarization into the pro-inflammatory M1 sub-type, thereby enhancing the body’s immune defense against eESCs. Moreover, erastin promotes the progression of ferroptosis in eESCs [23].

To evaluate the efficacy of this therapy mode, we established a mouse endometriosis model and treated the mice with IL-33-Ab and erastin. In the endometriosis model mice, we found that IL-33-Ab inhibited endometriosis development, and the combination of IL-33-Ab with erastin further amplified this effect. These findings imply that ferroptosis immunotherapy has the potential to serve as a therapeutic strategy for managing endometriosis.

Our study also has limitations. Due to delayed clinical visits, most of the samples collected are from patients with middle-stage to late-stage endometriosis. Further studies including patients in earlier stages are necessary to gain a more comprehensive understanding of endometriosis progression.

**Fig. 8:**
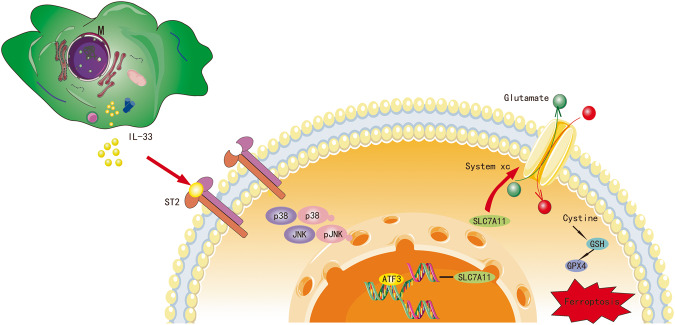
Cartoon illustration of IL-33/ST2 derived from macrophages inhibiting ferroptosis of ESCs via p38/JNK/ATF3/SLC7A11 pathway. Macrophage-derived IL-33 inhibits the ATF3-mediated reduction in SLC7A11 transcript levels via the P38/JNK pathway, ultimately resulting in protection against ferroptosis in eESCs.

In summary, we propose for the first time that IL-33 derived from macrophages can inhibit ferroptosis in eESCs by upregulating SLC7A11 expression through the p38/JNK/ATF3 pathway (Fig. 8). Our study provides a fresh perspective for understanding the pathological mechanism of endometriosis and developing novel treatment strategies.

## Materials and methods

### Clinical samples

This study was approved by the Ethical Committee of the Second Affiliated Hospital of Harbin Medical University (KY2016-040), and all patients provided informed written consent. The study recruited 26 women with endometriosis who were diagnosed by laparoscopy and histological analysis at the Second Affiliated Hospital of Harbin Medical University from March 2021 to April 2022. The normal endometriosis tissues were collected from 16 patients as the controls. Controls groups patients are whom underwent benign gynecological diseases including uterine prolapsehy, benign cervical lesions and without clinical symptom and sign of endometriosis or adenomyosis.

### Cell culture

Primary cells were isolated from endometriosis tissues using previously described protocols [11]. Briefly, the tissues were cut into 1 mm^3^ pieces and then digested using type IV collagenase (0.2% Sigma, USA) for 60 min at 37 °C. The primary cells were cultured in Dulbecco’s modified Eagle’s medium (DMEM) containing 15% fetal bovine serum (FBS; Biological Industries, Israel) and 1% penicillin‒streptomycin (Gibco, USA) at 37 °C with 5% CO_2_. Primary cells were identified using immunofluorescence staining (Supplementary Fig. 1).

The acute monocytic leukemia cell line (THP-1 cells), was purchased from ScienCell, and cultured in Roswell Park Memorial Institute 1640 (RPMI-1640) medium supplemented with 10% FBS and 1% penicillin‒streptomycin (Biological Industries, Israel). THP-1 cells were treated with PMA (200 nM; Sigma) for 48 h to induce cell polarization.

### Immunohistochemical staining (IHC)

The sections were incubated overnight and then immersed in xylene and ethanol for deparaffinization. Primary antibodies were incubated for overnight at 4 °C, followed by incubation with secondary antibodies for 20 min at ambient temperature (as listed in Table 1). The sections were then stained using DAB dye (CWBIO, Beijing, China) and hematoxylin. Finally, the slides were covered with cover slips.

**Table 1:** Details of antibody used in experiments.

Antigen	Catalog number	Dilution	Source	Species
IF				
IL-33	66235-1-Ig	1:200	Proteintech	Mouse
Vimentin	10366-1-AP	1:50	Proteintech	Rabbit
Cytokeratin 7	15539-1-AP	1:50	Proteintech	Rabbit
CD11b	66519-1-Ig	1:200	Proteintech	Mouse
CD68	28058-1-AP	1:200	Proteintech	Rabbit
WB				
β-actin	60008-1-Ig	1:20,000	Proteintech	Mouse
IL-33	66235-1-Ig	1:1000	Proteintech	Mouse
ST2	60112-1-Ig	1:20,000	Proteintech	Mouse
ACSL4	22401-1-AP	1:1000	Proteintech	Rabbit
SLC7A11	26864-1-AP	1:1000	Proteintech	Rabbit
GPX4	14432-1-AP	1:5000	Proteintech	Rabbit
ATF3	DF3110	1:1000	Affinity	Rabbit
JNK	66210-1-Ig	1:5000	Proteintech	Mouse
p-JNK	80024-1-RR	1:1000	Proteintech	Rabbit
p38	14064-1-AP	1:1000	Proteintech	Rabbit
p-p38	28796-1-AP	1:1000	Proteintech	Rabbit
GAPDH	10494-1-AP	1:5000	Proteintech	Rabbit
IHC				
IL-33	12372-1-AP	1:200	Proteintech	Rabbit
ST2	60112-1-Ig	1:200	Proteintech	Mouse
ATF3	DF3110	1:100	Affinity	Rabbit
ST2 (Mouse)	11920-1-AP	1:400	Proteintech	Rabbit
SLC7A11 (Mouse)	26864-1-AP	1:200	Proteintech	Rabbit

*WB* western blot, *IHC* immunohistochemistry, *IF* Immunofluorescence, *IL-33* interleukin-33, *IL1R-L1* ST2 interleukin receptor-like 1, *ACSL4* acyl-CoA synthetase long-chain family member 4, *SLC7A11* solute carrier family 7 member 11, *GPX4* glutathione peroxidase 4, *ATF3* activating transcription factor, *JNK* c-Jun N-terminal protein kinase, *p-JNK* phosphorylated c-Jun N-terminal protein kinase, *p38/p38 MAPK* p38 mitogen activated protein kinases, *p-p38* phosphorylated p38 mitogen activated protein kinases.

### Quantitative real-time PCR (RT-qPCR)

Total RNA was extracted from cells or tissues using TRIzol (Invitrogen, USA), isopropyl alcohol, chloroform, and 75% ethanol. Reverse transcription of RNA (500 ng) was accomplished using a cDNA synthesis kit according to the manufacturer’s protocol. Thereafter, cDNA (20 ng) was employed as a template for RT-qPCR using the Top Green qPCR SuperMix kit (TransGen Biotech, China). The primers were obtained from GENEWIZ (GENEWIZ, China) and are listed in Table 2.

**Table 2:** Sequences of primers used for RT-qPCR analysis.

Gene	Forward primer sequence	Reverse primer sequence
β-actin	TCCATGAAGTGACG	TACTCCTGCTTGCTGATCCACAC
IL-33	GATGGGAAGAAGGTG ATGGTG	TTG TGAAGGACGAAGAAGGC
ST2	CAACTGGACAGCACCTCTTG	GGTAATCACCTGCGTCCT
SLC7A11	CCCTTTGCTCTCATACCCATC	GACTTTCCTCTTCAGCTGCACTT
ATF3	GGAGTGCCTGCAGAAAGAGT	CCATTCTGAGCCCGGACAAT

*IL-33* interleukin-33, *IL1R-L1* ST2 interleukin receptor-like 1, *SLC7A11* solute carrier family 7 member 11, *ATF3* activating transcription factor, *qRT-PCR* reverse transcription and quantitative real-time PCR.

### Western blotting (WB)

Cells or tissues were lysed using RIPA lysis buffer and 1% PMSF (Beyotime Biotechnology, China). The protein samples were performed using a BCA protein assay kit (Beyotime Biotechnology, China). Samples were loaded into 10% SDS-PAGE gels (Epizyme, China) and then transferred onto PVDF membranes (Millipore, USA). The membrane was blocked using 5% skim milk powder solution for 2 h, after which it was incubated with primary antibodies (as listed in Table 1) overnight at 4 °C and then with secondary antibodies for 2 h at room temperature. The blots were visualized using ECL reagent (Epizyme, China).

### ELISA

Cell culture medium from each group was collected and centrifuged at 500 × *g*, for 5 min. Interleukin-33 (IL-33) concentrations in the culture media supernatant were measured via an ELISA kit (Proteintech, USA) in accordance with the manufacturer’s instructions.

### Immunofluorescence (IF)

Cells were fixed and subsequently blocked for 30 min using 5% goat serum albumin (Beyotime Biotechnology, China). The tissue sections were incubated overnight at 4 °C with specific primary antibodies (as listed in Table 1) and with fluorescent secondary antibodies for 1 h at room temperature. Fluorescence images were captured using a fluorescence microscope (Nikon, Tokyo, Japan).

### Cell viability assessment

Cell viability was measured by CCK-8 reagent following the manufacturer’s instructions (Beyotime Biotechnology, China). The absorbance readings were taken at 450 nm using a plate reader (Bio-Rad).

### Colony formation assay

Cells were subjected to 7 day of culture subsequently, staining was performed for 30 min using crystal violet dye. Imaging was carried out using an iPhone 12, and ImageJ software was then utilized to analyze the obtained data.

### Transwell migration assay

Cells were seeded at a density of 1×10^4^/mL in the upper chamber of the 8 μm transwell insert utilizing 100 μL of serum-free DMEM. The lower chamber was filled with 600 μL of DMEM containing 15% FBS. After a 24-h incubation, the cells were fixed and stained.

### Wound healing assay

We created a scratch in the cell monolayer with a 200 μL pipette. We recorded images of the scratch area under a microscope immediately after creating the scratch and at 24 and 48 h later.

### Cell transfection

We transfected cells with small interfering RNA (siRNA) using the Lipofectamine 3000 transfection kit (Invitrogen, USA) according to the manufacturer’s instructions. Ribobio (Ribobio, China) synthesized all specific siRNAs and siRNA controls. The target sequences of the siRNAs were as follows: siRNA, 5ʹ-TTCTCCGAACGTGTCACGT-3ʹ; siST2-1, 5ʹ-TCTAAUGUCACTAAAUAACUT-3ʹ; siST2-2, 5ʹ-GCGAAUGUCACCAUAUAUATT-3ʹ; siST2-3, 5ʹ-GCCCATGUCATTAAAUAUCAT-3ʹ; siSLC7A11-1, 5ʹ-CCGGCCTGTCACTATTT-3ʹ; siSLC7A11-2, 5ʹ-GGAAGAGATTCAAGTATTA-3ʹ; siSLC7A11-3, 5ʹ-GGAGCTTTCTCGAGAAAG-3ʹ; siATF3-1, 5ʹ-CCGCCTTTCATCTGGATTCTA-3ʹ; and siATF3-2, 5ʹ-GCTGAACTGAAGGCTCAGATT-3ʹ. siATF3-3, 5ʹ-GCTGCAAAGTGCCGAAACA-3ʹ.

### Measurement of intracellular iron levels

We used the FerroOrange kit (Dojindo, Japan) to detect intracellular Fe2+ levels in eESCs. The cells were incubated with serum-free medium containing 1 μM FerroOrange reagent at 37 °C and 5% CO_2_ for 30 min. We obtained fluorescence images of the cells using a confocal microscope (Nikon, Tokyo, Japan).

### Lipid peroxidation determination

To quantify the levels of lipid peroxidation, we used the LiperFluo and MDA Assay Kit from Dojindo (Dojindo, Japan) and Beyotime Biotechnology (Beyotime, China), respectively. For the LiperFluo assay, we added LiperFluo reagent (5 μM) diluted in DMEM to the treated cells and incubated them for 30 min at 37 °C and 5% CO_2_. We evaluated lipid peroxidation levels using fluorescence microscopy (Nikon, Tokyo, Japan) by capturing photographs of the cells. For the MDA assay, cell lysis buffer for Western blotting and IP (Beyotime Biotechnology, China) was used to lyse the cells on ice for 30 min. The absorbance was then measured at 532 nm using a plate reader in line with the manufacturer’s instructions.

### Measurement of GSH levels

We measured intracellular GSH levels in the treated cells using a GSH assay kit from Solarbio (Solarbio, Beijing, China). We lysed the cells entirely by performing four consecutive freeze-thaw cycles. We then mixed the cell lysate with the GSH reagent and measured the OD value of the resulting mixture at 412 nm using a plate reader.

### Transmission electron microscopy (TEM)

Samples were fixed with 2.5% glutaraldehyde (Servicebio, China) following a previously established protocol. After dehydration, we cut the samples into thin slices, which were then stained with uranyl acetate and lead citrate for contrast enhancement. Finally, we captured TEM images of the samples using a Hitachi TEM system (Japan).

### ChIP

We used the DNA ChIP Assay Kit (Beyotime Biotechnology, China) to immunoprecipitate DNA according to the manufacturer’s instructions. Collected cell sample fragments were diluted in ChIP dilution buffer, followed by incubation with Protein A + G Agarose/Salmon Sperm at 4 °C for 30 min. We later incubated the samples with anti-ATF3 antibody (Affinity, DF3110) or normal rabbit IgG overnight at 4 °C. We purified the samples using a DNA purification kit from Beyotime Biotechnology (China). Finally, we used RT-qPCR to quantify the predicted DNA sequences in the immunoprecipitated samples. The primers used were as follows: SLC7A11, forward 5’-TTGAGCAACAAGCTCCTCCT-3’, reverse 5’-CAAACCAGCTCAGCTTCCTC-3’.

### Mouse endometriosis model

Female C57BL/6 mice (7 weeks old) were randomly sorted into four groups (*n* = 6); the endometriosis group, endometriosis+erastin group, endometriosis+IL-33-Ab group, and endometriosis+erastin+IL-33-Ab group. The endometriosis models were established as described previously [11]. Briefly, the uterus from an estradiol (0.2 ml/mouse)-stimulated donor mouse, was minced into 1 mm^3^ fragments and immediately injected subcutaneously into the peritoneal cavity of two recipient mice. The abdominal cavity of mice was subcutaneously injected with 300 μL of erastin (20 mg/kg) (MCE, Shanghai, China) alone or combined with 50 μg IL-33-Ab (R&D Systems, USA), or normal saline (Fig. 7A). On Day 10 postsurgery, all mice were sacrificed. The lesion volume was calculated using the Formula *V* = 1/2*Aa*^2^; *A*: long radius, *a*: short radius. Ethical approval for all animal research was granted by the Institutional Animal Research Ethics Committee of Harbin Medical University.

### Statistical analysis

Each experiment was conducted independently, with a total of three replicates. Statistical was performed using GraphPad Prism 8 (San Diego, USA), and the data are presented as the mean with standard deviation (SD). Student’s *t* test, one-way ANOVA, and two-way ANOVA were used to compare data in different experimental groups. Statistical significance was determined as *p* < 0.05. Non-significant differences were designated as “ns” (*p* ≥ 0.05), whereas ****p < 0.0001, ***p < 0.001, **p < 0.01, and *p < 0.05 represent significant differences.

## Supplementary information

Supplementary Fig. 1 Cell identification of ectopic endometrial stromal cells (eESCs) and normal endometrial stromal cells (nESCs) by immunofluorescence (IF).

Supplementary Fig. 2 ELISA assays were used to measure the concentration of IL-33 in cell medium.

Supplementary Fig. 3 Representative immunofluorescence (IF) images of ST2 (red) in nESCs and eESCs.

Supplementary Fig. 4 Cell identification of macrophages induced by PMA.

Supplementary Fig. 5 Quantitative RT-PCR (RT-qPCR) was used to determine the relative levels of IL-33 mRNA in eESCs.

Supplementary Fig. 6 The statistic graphs for Figure 2E and 2F in the manuscript.

Supplementary Fig. 7 Pearson’s test was used to analyze the relationship between the expression levels of IL-33 and GPX4 mRNA in EC tissues.

Supplementary Fig. 8 Quantitative RT-PCR (RT-qPCR) was used to determine the relative levels of SLC7A11 mRNA in eESCs.

Supplementary Fig. 9 Quantitative RT-PCR (RT-qPCR) was used to determine the relative levels of ATF3 mRNA in eESCs.

Supplementary Figure legends

Original Data File 1

Original Data File 2

aj-checklist.

## Data Availability

The original contributions presented in the study are included in the article/Supplementary Material, further inquiries can be directed to the corresponding author.
