# Correction: Macrophages originated IL-33/ST2 inhibits ferroptosis in endometriosis via the ATF3/SLC7A11 axis

**DOI:** 10.1038/s41419-025-07484-5

**Published:** 2025-03-27

**Authors:** Qiong Wu, Zongwen Liang, Jing Jiang, Xiaoming Feng, Jinming Liu, Zongfeng Zhang, Honglin Wang, Ning Wang, Yanling Gou, Zhi Li, Yingying Cao

**Affiliations:** 1 Department of Obstetrics and Gynecology, Second Affiliated Hospital of Harbin Medical University, 150086 Harbin, China; 2 Academy of Agriculture and Food Science and Technology, HeiLongJiang Agricultural Engineearing Vocational College, Harbin, China

**Keywords:** Apoptosis, Interleukins, Immunoproliferative disorders, Cell death and immune response, DNA metabolism

Correction to: *Cell Death and Disease* 10.1038/s41419-023-06182-4, published online 11 October 2023

In this article the following mistakes have been identified:

1. JNK Bands Error: During the image preparation process, the p-p38 (phosphorylated p38) band was mistakenly labeled as JNK. This mislabeling led to the incorrect representation of the displayed band, potentially causing confusion in data interpretation. The authors have corrected this by replacing the mislabeled band with the appropriate JNK band images that accurately correspond to the p-JNK band images.
2. Statistical Chart Discrepancy: A discrepancy was identified between the statistical chart and the gel images in Figure 6B, which arose from an oversight during figures preparation. The authors have recalculated the grayscale values from the gel images and conducted a new quantitative analysis. The revised statistical data has been incorporated into the updated figure.

These corrections do not alter the results or conclusions of this study. All authors have reviewed and agreed to this corrigendum and express their gratitude to the Editor of Cell Death & Disease for providing the opportunity to address these issues. Finally, the authors sincerely apologize to the readership for any inconvenience these errors may have caused.

Original Figure 6
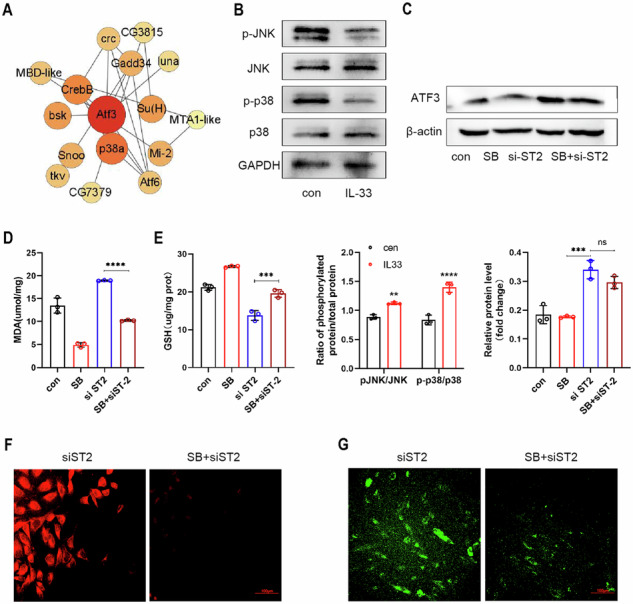


Corrected Figure 6
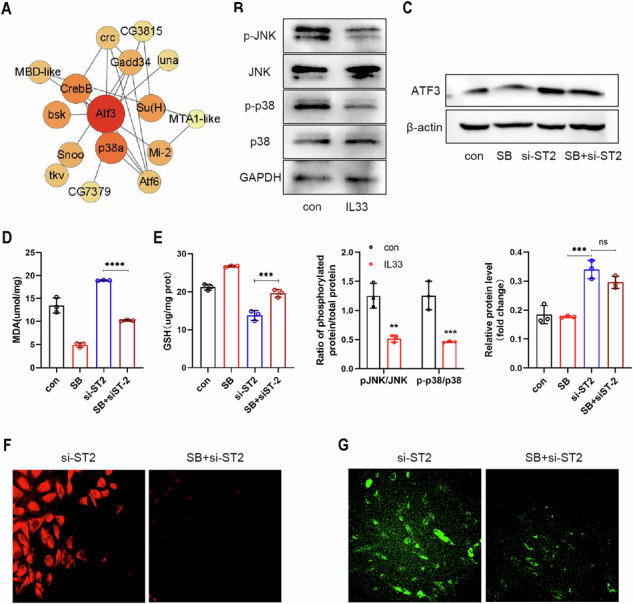


We further investigated the effects of rIL-33 on the P38 MAPK/JNK signaling pathway. Western blot images showed that after treatment with recombinant protein IL-33, the ratio of phosphorylated modified JNK (p-JNK) to total JNK protein in eESCs was downregulated and the ratio of phosphorylated p38 (p-p38) to total p38 protein is downregulated. Intriguingly, our findings revealed that treatment with rIL-33 suppressed the phosphorylation of both P38 MAPK and JNK (Fig. 6B).

The original article has been corrected.

