# Supplementary figures and images for: Macrophages originated IL-33/ST2 inhibits ferroptosis in endometriosis via the ATF3/SLC7A11 axis
Source: Cell Death Dis. 2023 Oct 11;14(10):668. doi: 10.1038/s41419-023-06182-4 (PMC10564909; doi:10.1038/s41419-023-06182-4)

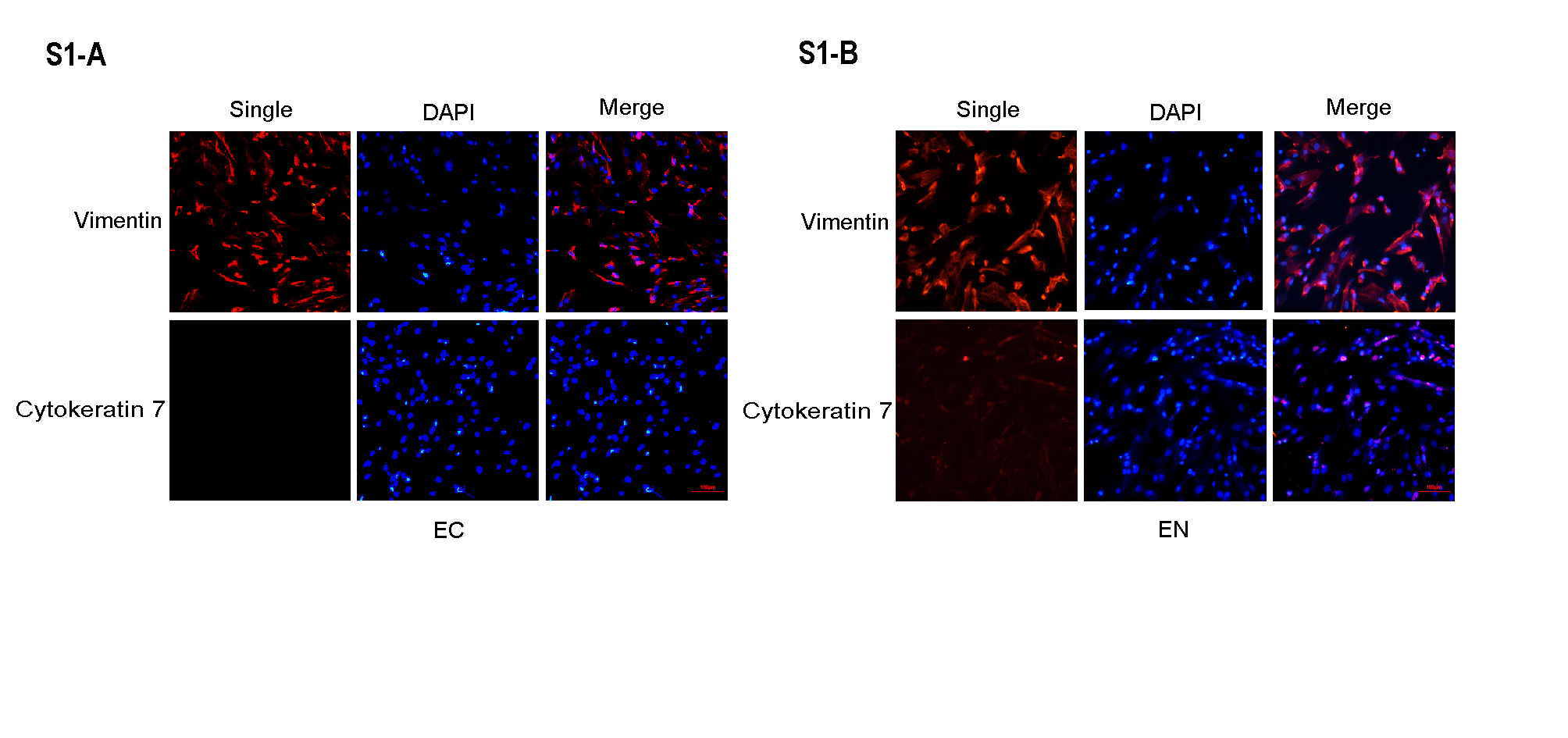

Supplement: Supplementary file 1 — Supplementary Fig. 1 Cell identification of ectopic endometrial stromal cells (eESCs) and normal endometrial stromal cells (nESCs) by immunofluorescence (IF). [file 41419_2023_6182_MOESM1_ESM.tif]

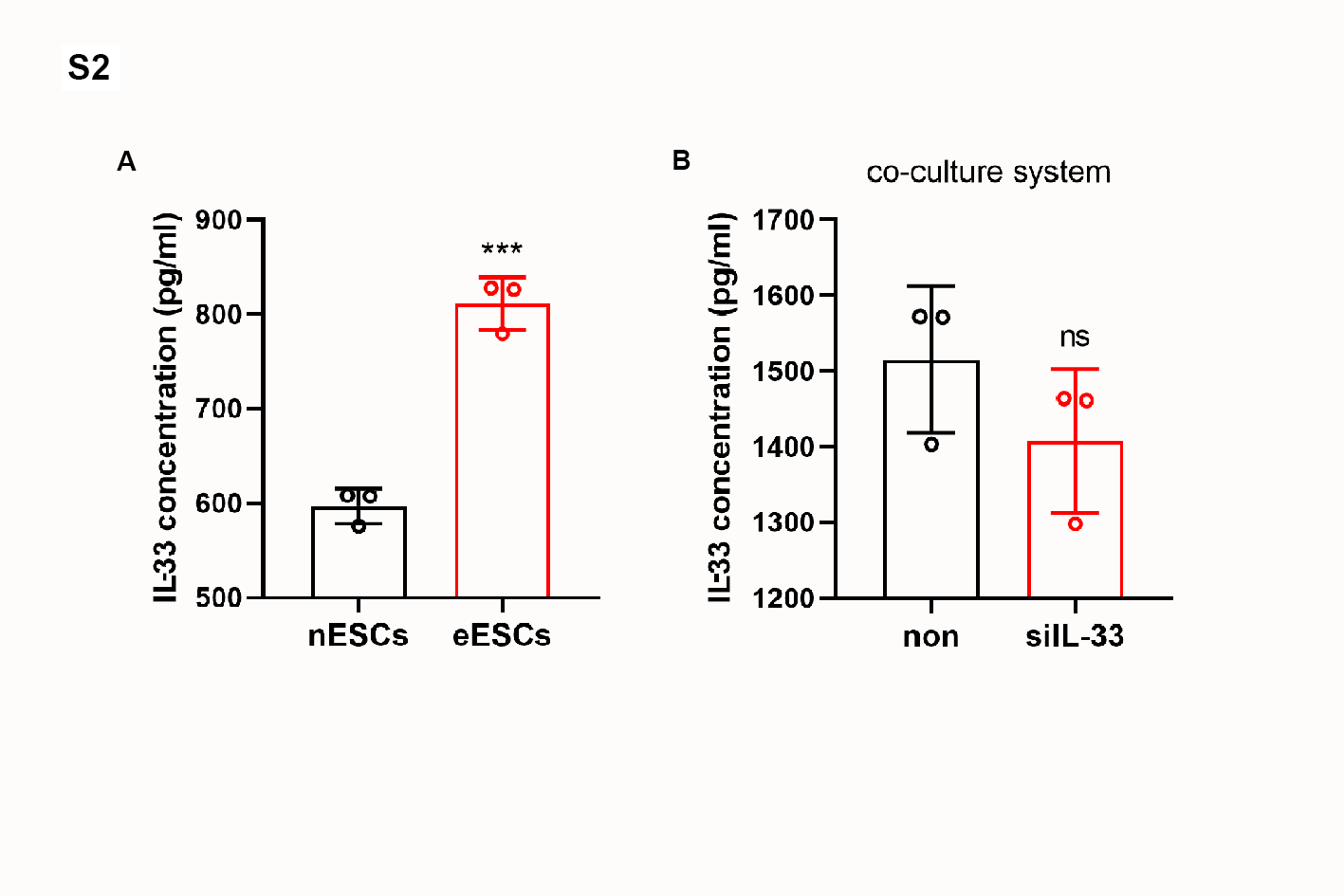

Supplement: Supplementary file 2 — Supplementary Fig. 2 ELISA assays were used to measure the concentration of IL-33 in cell medium. [file 41419_2023_6182_MOESM2_ESM.tif]

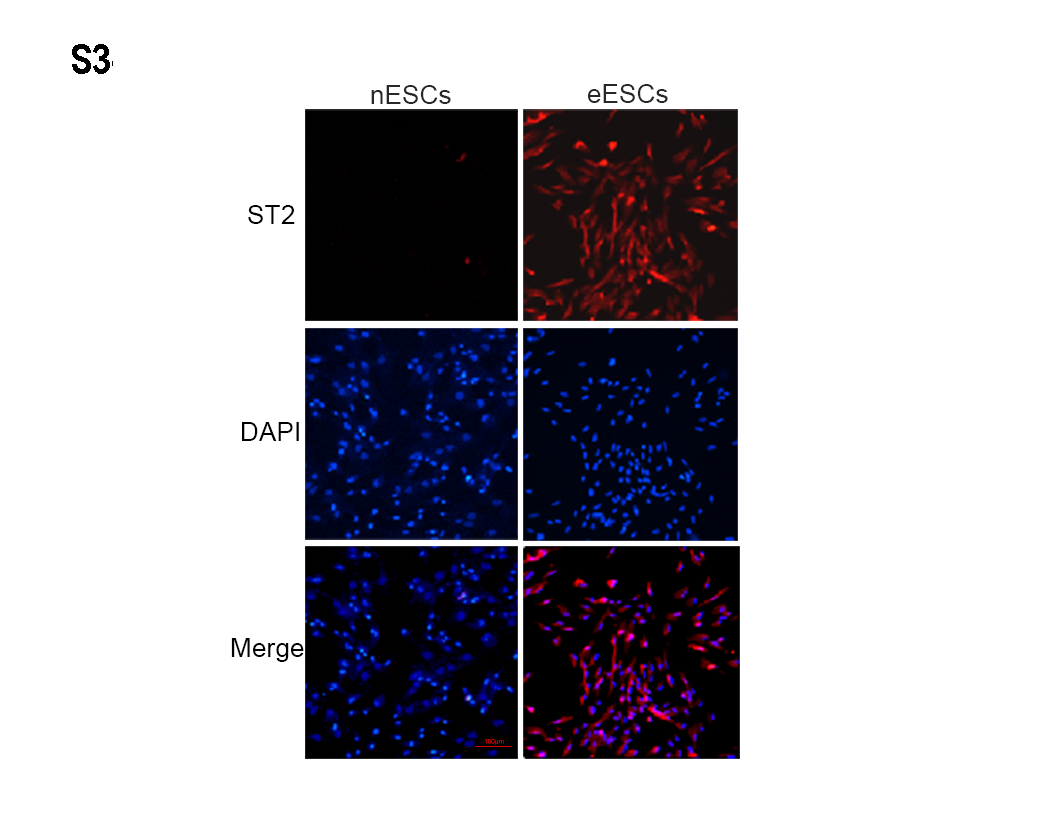

Supplement: Supplementary file 3 — Supplementary Fig. 3 Representative immunofluorescence (IF) images of ST2 (red) in nESCs and eESCs. [file 41419_2023_6182_MOESM3_ESM.tif]

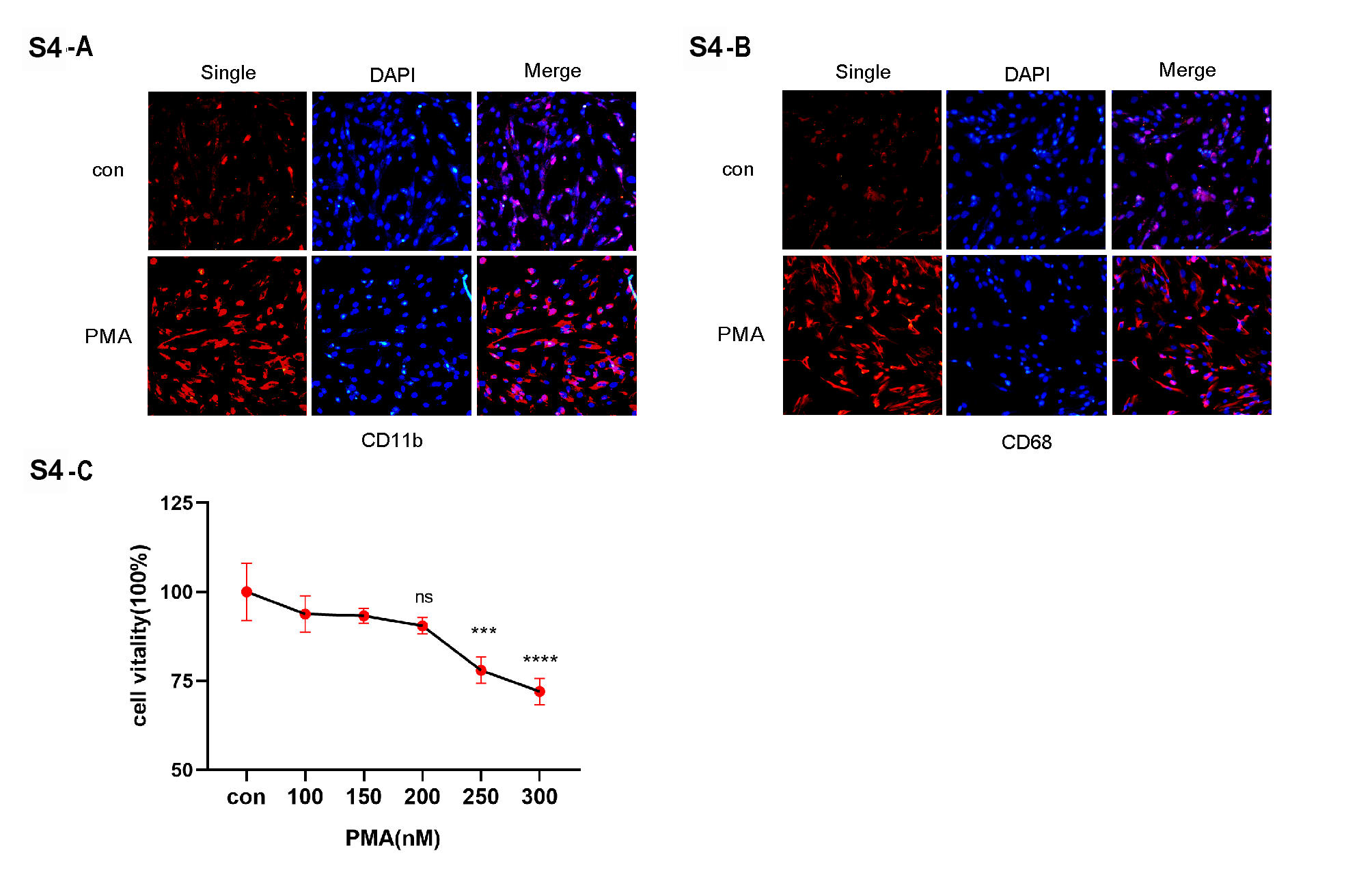

Supplement: Supplementary file 4 — Supplementary Fig. 4 Cell identification of macrophages induced by PMA. [file 41419_2023_6182_MOESM4_ESM.tif]

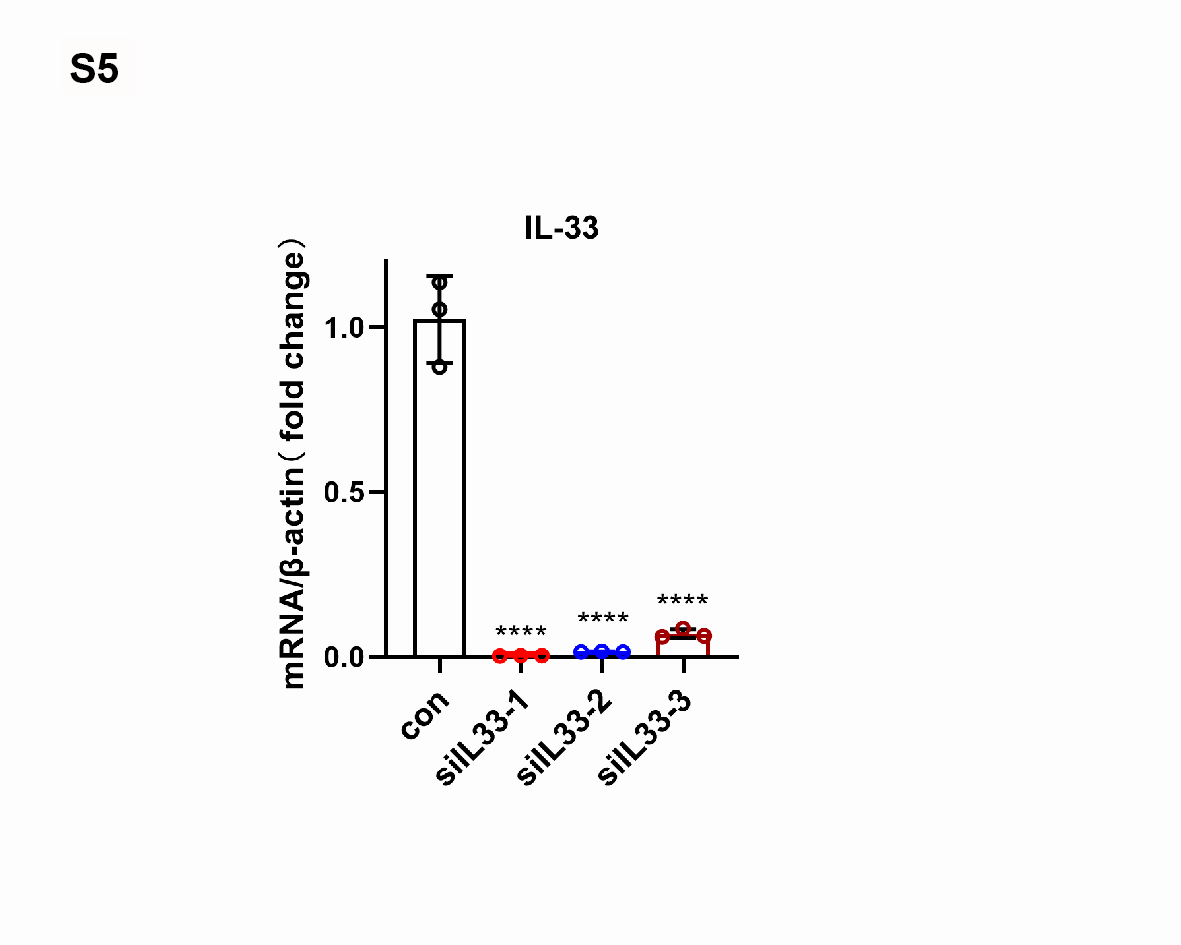

Supplement: Supplementary file 5 — Supplementary Fig. 5 Quantitative RT-PCR (RT-qPCR) was used to determine the relative levels of IL-33 mRNA in eESCs. [file 41419_2023_6182_MOESM5_ESM.tif]

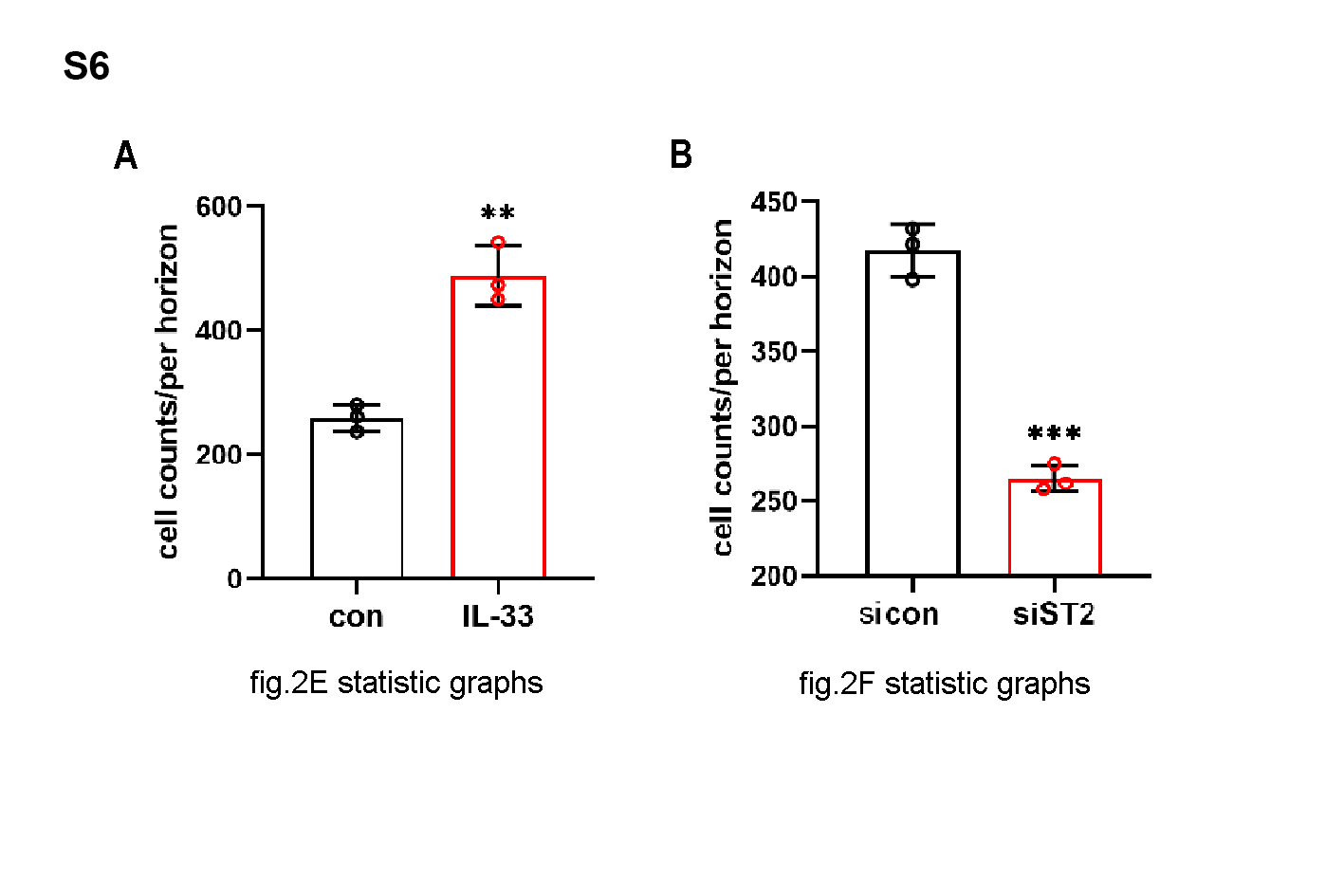

Supplement: Supplementary file 6 — Supplementary Fig. 6 The statistic graphs for Figure 2E and 2F in the manuscript. [file 41419_2023_6182_MOESM6_ESM.tif]

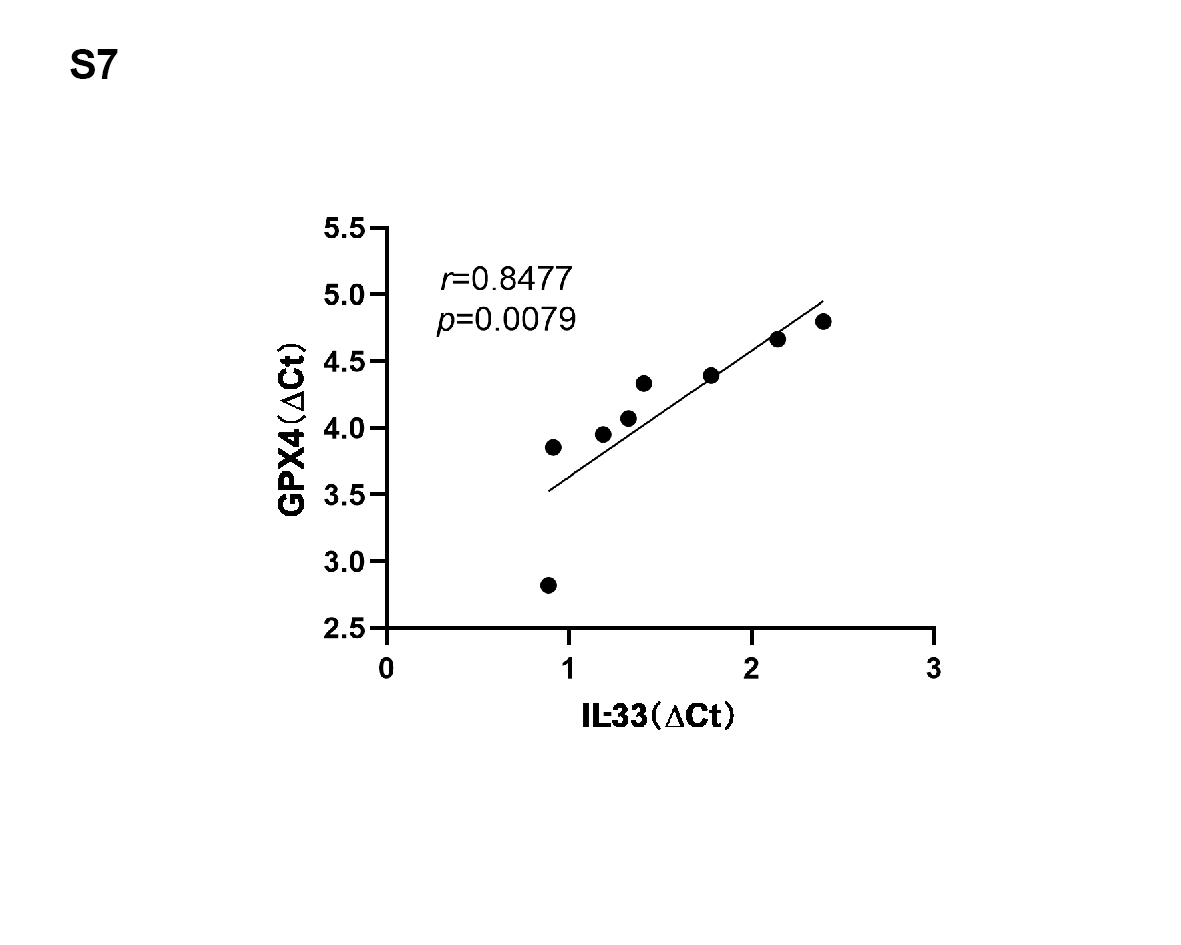

Supplement: Supplementary file 7 — Supplementary Fig. 7 Pearson’s test was used to analyze the relationship between the expression levels of IL-33 and GPX4 mRNA in EC tissues. [file 41419_2023_6182_MOESM7_ESM.tif]

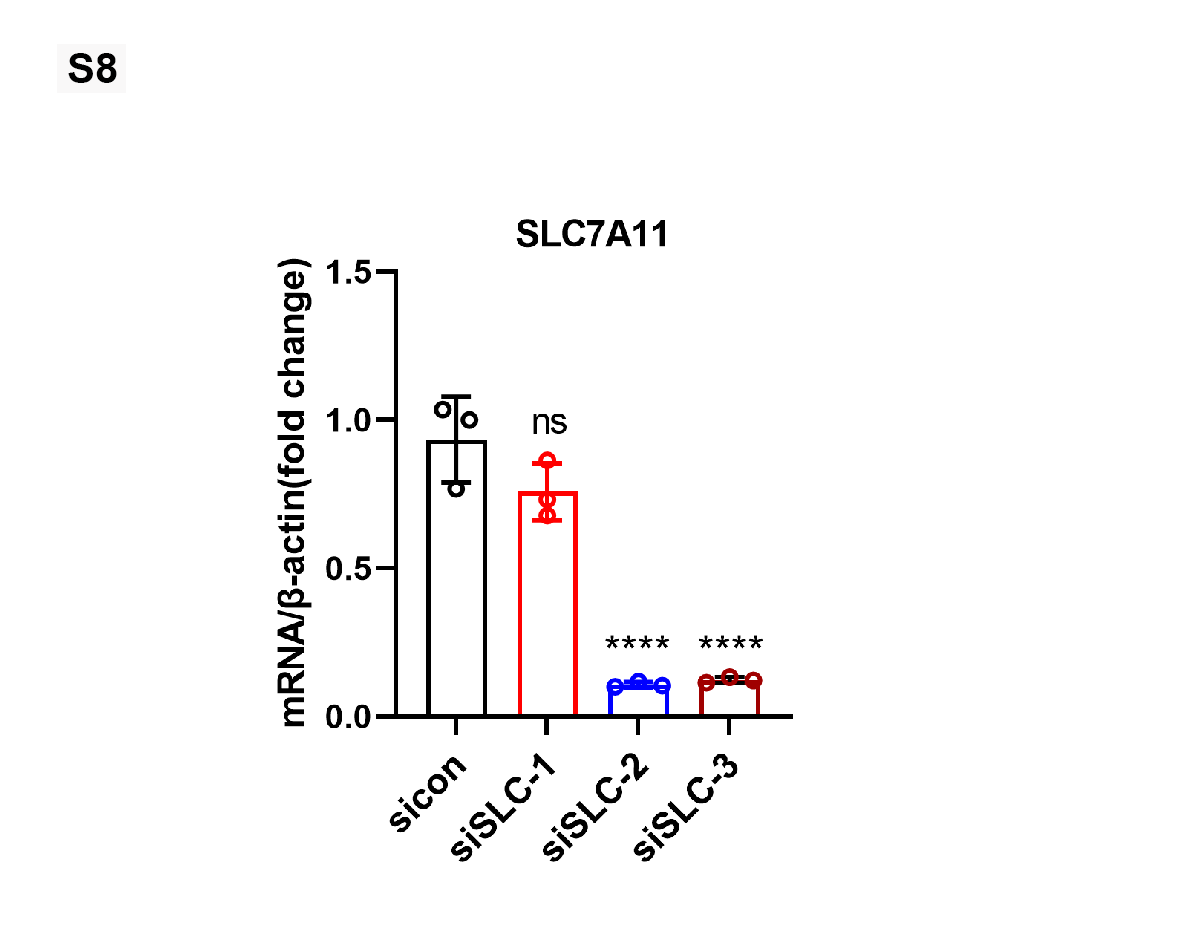

Supplement: Supplementary file 8 — Supplementary Fig. 8 Quantitative RT-PCR (RT-qPCR) was used to determine the relative levels of SLC7A11 mRNA in eESCs. [file 41419_2023_6182_MOESM8_ESM.tif]

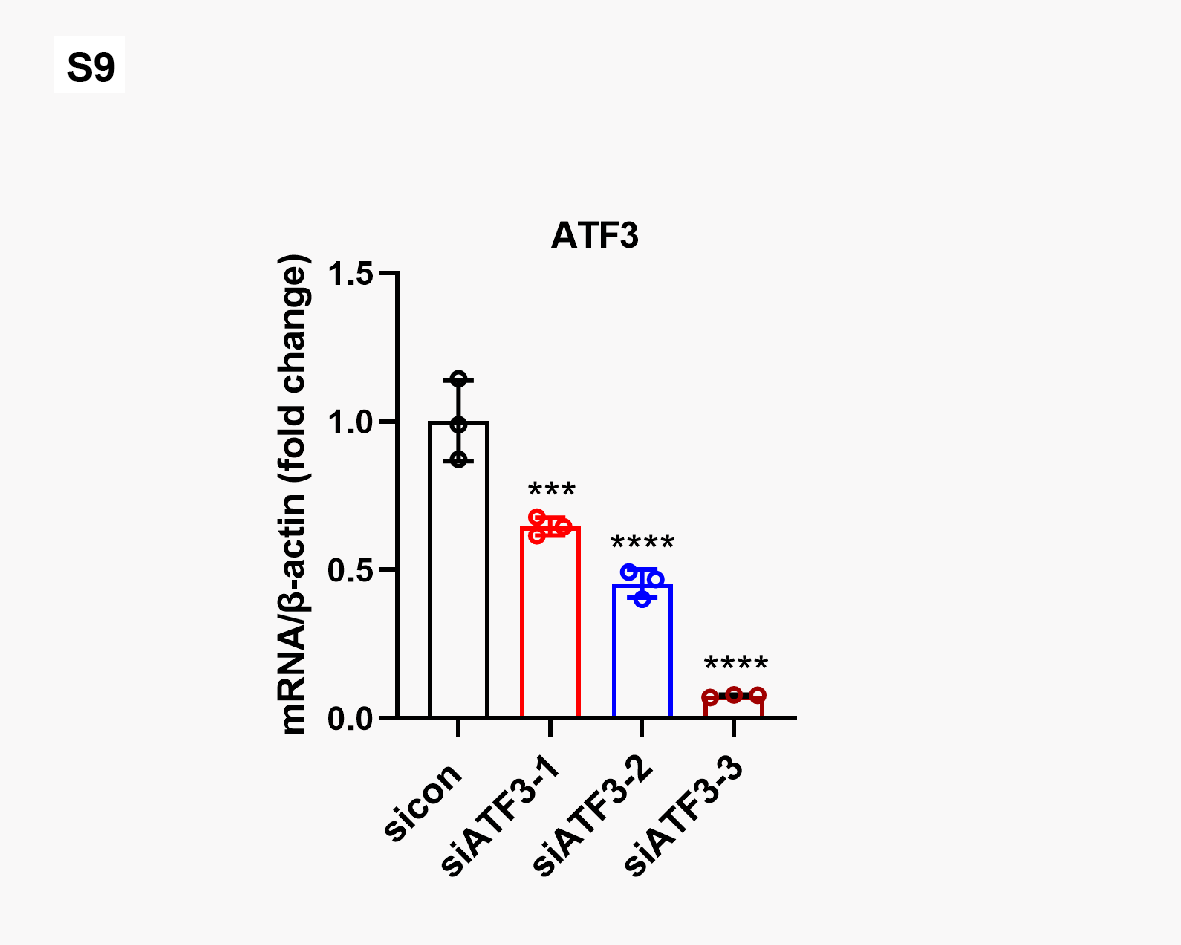

Supplement: Supplementary file 9 — Supplementary Fig. 9 Quantitative RT-PCR (RT-qPCR) was used to determine the relative levels of ATF3 mRNA in eESCs. [file 41419_2023_6182_MOESM9_ESM.tif]

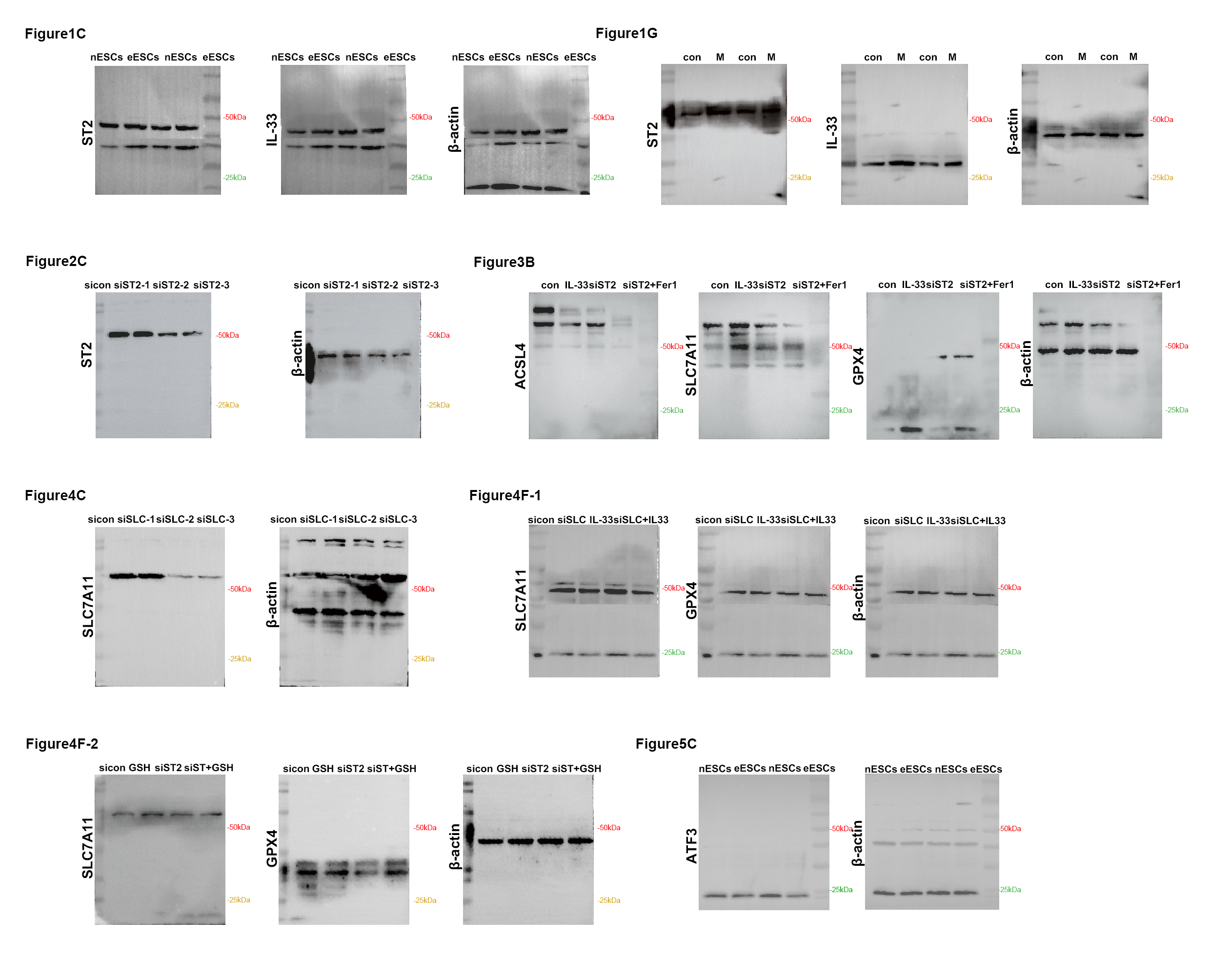

Supplement: Supplementary file 11 — Original Data File 1 [file 41419_2023_6182_MOESM11_ESM.tif]

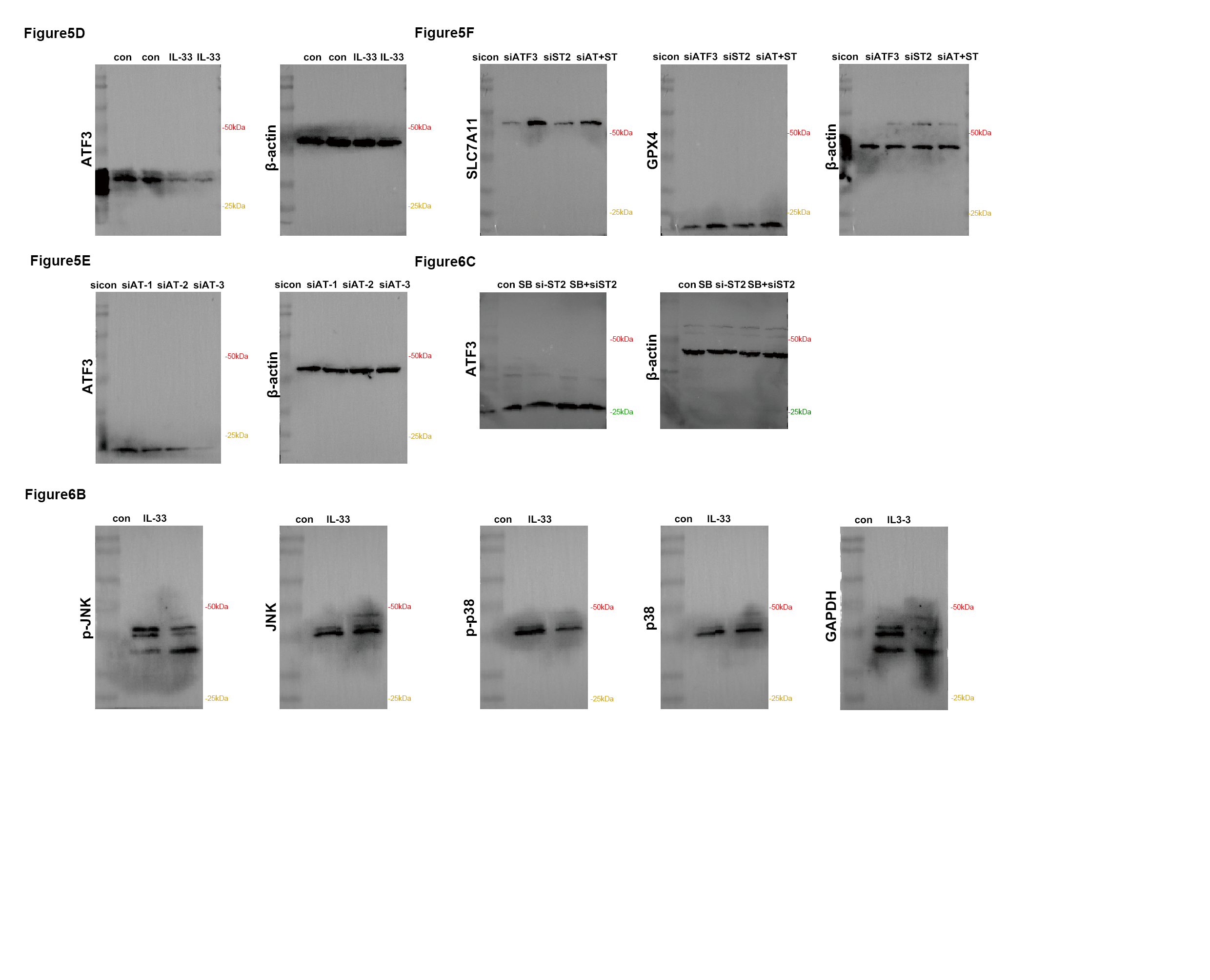

Supplement: Supplementary file 12 — Original Data File 2 [file 41419_2023_6182_MOESM12_ESM.tif]
