# Supplementary material for: Macrophages originated IL-33/ST2 inhibits ferroptosis in endometriosis via the ATF3/SLC7A11 axis
Source: Cell Death Dis. 2023 Oct 11;14(10):668. doi: 10.1038/s41419-023-06182-4 (PMC10564909; doi:10.1038/s41419-023-06182-4)
Supplement: Supplementary file 10 — Supplementary Figure legends [file 41419_2023_6182_MOESM10_ESM.docx]

**Supplementary Figure legends:**

**Supplementary Fig. 1** (A, B) Cell identification of ectopic endometrial stromal cells (eESCs) and normal endometrial stromal cells (nESCs) by immunofuorescence (IF). Vimentin or Cytokeratin 7 (red), Nuclei stained with DAPI (blue). (original magnification 200×).

**Supplementary Fig. 2** (A) ELISA assay was used to measure the concentration of IL-33 in cell medium of eESCs or nESCs. (B) ELISA assay was used to measure the concentration of IL-33 in the medium of macrophages and eESCs co-culture system with or without IL-33 knockdown eESCs, Data are presented as the mean ± SD, n=3 independent experiments. Statistical analysis was performed using Student’s t test. ****p*<0.001.

**Supplementary Fig. 3** Representative immunofuorescence (IF) images of ST2 (red) in nESCs and eESCs, Nuclei were stained with DAPI (blue). (original magnification 200×).

**Supplementary Fig. 4** (A, B) Cell identification of macrophages induced by PMA. CD11b or CD68 (red), Nuclei stained with DAPI (blue). (original magnification 200×). (C) Cell viability of THP-1 cells treated with PMA was detected by CCK-8 assay.

**Supplementary Fig. 5** Quantitative RT-PCR (RT-qPCR) was used to determine the relative levels of IL-33 mRNA in eESCs transfected with siIL-33 (50nM), sicon (50nM) for 48 hours.

**Supplementary Fig. 6** (A, B) The statistic graphs for Figure 2E and 2F in the manuscript. Statistical analysis was performed using Student’s t test. ****p*<0.001, ***p*<0.01.

**Supplementary Fig. 7** Pearson’s test was used to analyze the relationship between the expression levels of IL-33 and GPX4 mRNA in EC tissues (n=8).

**Supplementary Fig. 8** Quantitative RT-PCR (RT-qPCR) was used to determine the relative levels of SLC7A11 mRNA in eESCs transfected with siSLC7A11 (50nM), sicon (50nM) for 48 hours.

**Supplementary Fig. 9** Quantitative RT-PCR (RT-qPCR) was used to determine the relative levels of ATF3 mRNA in eESCs transfected with siATF3 (50nM), sicon (50nM) for 48 hours.
